# Cascade enzymatic synthesis of a statin side chain precursor – the role of reaction engineering in process optimization†

**DOI:** 10.1039/d4ra01633e

**Published:** 2024-07-04

**Authors:** Martina Sudar, Nevena Milčić, Morana Česnik Katulić, Anna Szekrenyi, Karel Hernández, Melinda Fekete, Rainer Wardenga, Maja Majerić Elenkov, Yuyin Qi, Simon Charnock, Đurđa Vasić-Rački, Wolf-Dieter Fessner, Pere Clapés, Zvjezdana Findrik Blažević

**Affiliations:** a University of Zagreb Faculty of Chemical Engineering and Technology Savska c. 16 HR-10000 Zagreb Croatia msudar@fkit.unizg.hr zfindrik@fkit.unizg.hr +385 1 4597 133 +385 1 4597 157 +385 1 4597 101; b Technische Universität Darmstadt Peter-Grünberg-Straße 4 64287 Darmstadt Germany; c Institute of Advanced Chemistry of Catalonia, Biotransformation and Bioactive Molecules Group, IQAC-CSIC Jordi Girona 18-26 08034 Barcelona Spain; d Enzymicals AG Walther-Rathenau-Straße 49b 17489 Greifswald Germany; e piCHEM Forschungs-und Entwicklungs GmbH Parkring 3 8074 Raaba-Grambach Austria; f Ruđer Bošković Institute Bijenička cesta 54 HR-10 000 Zagreb Croatia; g Prozomix Ltd Haltwhistle Northumberland NE49 9HA UK

## Abstract

Statins are an important class of drugs used to lower blood cholesterol levels and are often used to combat cardiovascular disease. In view of the importance of safe and reliable supply and production of statins in modern medicine and the global need for sustainable processes, various biocatalytic strategies for their synthesis have been investigated. In this work, a novel biocatalytic route to a statin side chain precursor was investigated in a one-pot cascade reaction starting from the protected alcohol *N*-(3-hydroxypropyl)-2-phenylacetamide, which is oxidized to the corresponding aldehyde in the first reaction step, and then reacts with two equivalents of acetaldehyde to form the final product *N*-(2-((2*S*,4*S*,6*S*)-4,6-dihydroxytetrahydro-2*H*-pyran-2-yl)ethyl)-2-phenylacetamide (phenylacetamide-lactol). To study this complex reaction, an enzyme reaction engineering approach was used, *i.e.* the kinetics of all reactions occurring in the cascade (including side reactions) were determined. The obtained kinetic model together with the simulations gave an insight into the system and indicated the best reactor mode for the studied reaction, which was fed-batch with acetaldehyde feed to minimize its negative effect on the enzyme activity during the reaction. The mathematical model of the process was developed and used to simulate different scenarios and to find the reaction conditions (enzyme and coenzyme concentration, substrate feed concentration and flow rate) at which the highest yield of phenylacetamide-lactol (75%) can be obtained. In the end, our goal was to show that this novel cascade route is an interesting alternative for the synthesis of the statin side chain precursor and that is why we also calculated an initial estimate of the potential value addition.

## Abstract 2

Statins are an important class of drugs used to lower blood cholesterol levels and are often used to combat cardiovascular disease.

## Graphical abstract

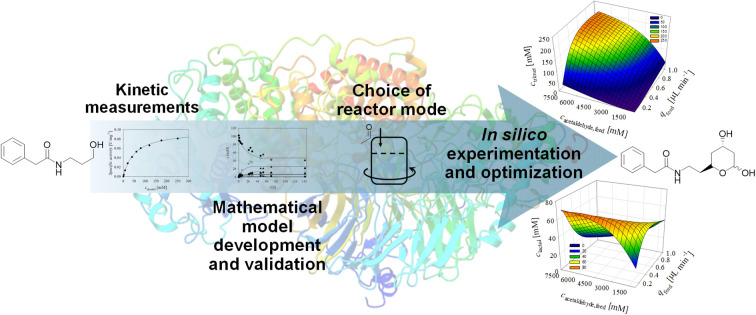

## Introduction

The chemical industry is an industry that qualifies modern life by providing advanced medicines, drugs, materials *etc.*, but it is also perceived by the public as one of the major sources of pollution.^1,2^ This dual perception drives the global need for an initiative to make industrial processes more environmentally friendly. Scientists from different disciplines are trying to contribute to the greening of the chemical industry through various measures. In the last decade, many large pharmaceutical companies have moved towards using environmentally friendly processes in the discovery, development and manufacture of pharmaceuticals, allowing cost reductions and better environmental performance.^3–5^ The environmental friendliness of a process can be quantified through calculation of its *E*-factor,^6^*i.e.*, by defining the amount of waste generated per kg of product. This key figure makes it possible to compare different processes, facilitating the selection of the best one. The pharmaceutical and fine chemical industries are among the sectors with the highest waste generation, with E-factors of 25–100 and 5–50, respectively.^6^ These industries are therefore a problematic burden on the environment, highlighting the need for substantial changes in these sectors. Biocatalysis has been widely used in industry for decades, in particular due to its exquisite selectivity which eliminates the need for protecting groups and shortens entire reaction schemes.^4,7,8^ In many cases, enzymes represent a minimal environmental and economic burden,^9–11^ in particular due to the potential today to optimize their properties through enzyme engineering,^10,12,13^ and the possibility to reuse them through immobilization.^14,15^ The use of enzymes can have many advantages,^8,16,17^*e.g.*, the acceptance of mild reaction conditions in aqueous systems, high selectivity and the possibility of producing lower amounts of waste and by-products. Probably the greatest advantage of using enzymes is their selectivity and ability to efficiently control chirality during drug synthesis.^17,18^ Therefore, enzymatic synthesis is a proven alternative to the traditional, chemical synthesis. Chemo-catalysis often operates under more rigorous conditions, including high temperatures and pressures, and can enable a broader range of reactions compared to enzyme catalysis. Enzymes have the remarkable ability to accelerate chemical reactions under mild conditions, enabling the synthesis of complex molecules with extreme precision. Nevertheless, it must be pointed out that although biocatalysis is considered environmentally friendly, this must be evaluated on a process-by-process basis.^8,19–25^ One of the ways to improve the environmental impact of the process is to couple reactions into multistep syntheses without isolating the intermediate products, also known as cascade reactions.^26,27^ This way of producing chemicals has attracted considerable interest due to the enormous potential benefits. In particular, the number of references dealing with the design and development of cascade reactions has increased dramatically over the last decade.^26,28–31^ Cascade reactions use fewer chemicals, especially due to the elimination of solvent-intensive processing of intermediates; they can be carried out in one reactor, thus reducing the number of process units; they can be used to shift the reaction equilibrium towards the desired products; they can work well with unstable intermediates that are produced and consumed *in situ*.^32,33^ Despite these advantages, finding optimal conditions for cascade reactions requires considerable effort, and yields and productivities can still be suboptimal. The complexity of such systems, with their numerous variables and dependencies, can be effectively managed using an engineering approach, such as kinetic modelling^34,35^ or design of experiments.^36^ In that manner, a vast variable space can be investigated by performing *in silico* experiments, different reactor set-ups and process conditions to be explored.^37–39^

Statins are a class of medication used for lowering the level of cholesterol in the bloodstream.^40^ They work by inhibiting 3-hydroxy-3-methylglutaryl-coenzyme A (HMG-CoA) reductase, an enzyme involved in the biosynthesis of cholesterol.^41^ Cardiovascular disease remains the leading cause of death years of life lost to disability worldwide,^42^ so statin prescribing rates continue to rise in response to stricter regulations to maintain healthy cholesterol levels and the constant expansion of patient eligibility criteria.^43^ Considering the importance of safe and reliable statin supply and production in modern medicine, as well as the global need for sustainable processes, various biocatalytic strategies for 3,5-dihydroxy acid side chain syntheses with two chiral centres have been investigated, including enzymes from different families such as alcohol dehydrogenases, aldolases, nitrilases, lipases, ketoreductases, halohydrin dehalogenases *etc.*^44–52^

**Scheme 1:**
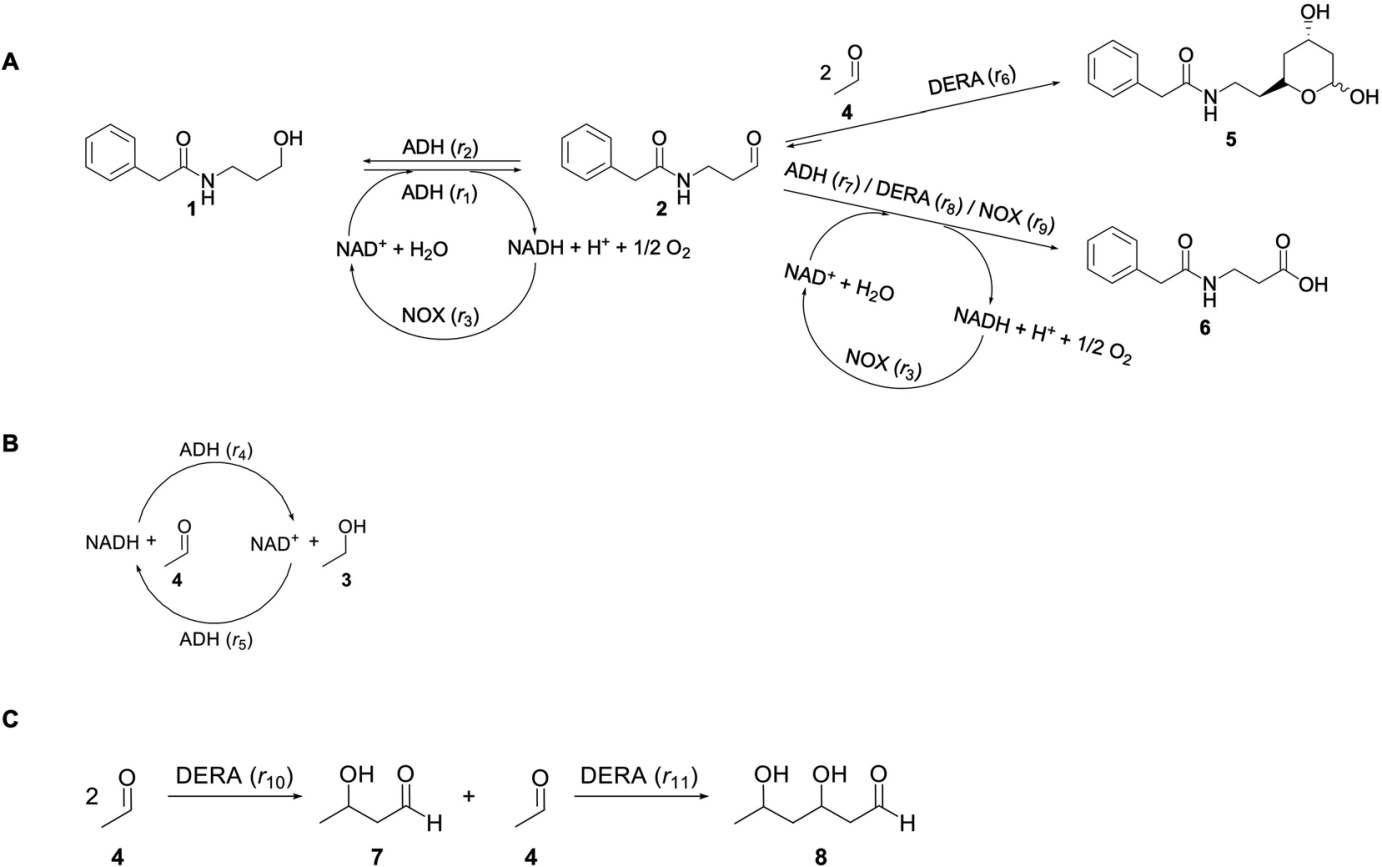
(A) Reaction scheme, (B) coenzyme regeneration catalyzed by ADH *via* acetaldehyde reduction, (C) acetaldehyde dimerization and trimerization (1 – *N*-(3-hydroxypropyl)-2-phenylacetamide (alcohol), 2 – *N*-(3-oxopropyl)-2-phenylacetamide (aldehyde), 3 – ethanol, 4 – acetaldehyde, 5 – *N*-(2-((2*R*,4*R*)-4,6-dihydroxytetrahydro-2*H*-pyran-2-yl)ethyl)-2-2-phenylacetamide (lactol), 6 – *N*-phenylacetyl-β-alanine (acid), 7 – 3-hydroxybutanal (dimer), 8 – 3,5-dihydroxyhexanal (trimer)).

In this paper a novel approach toward the precursor of the chiral statin side chains was investigated using an enzymatic cascade reaction starting from *N*-(3-hydroxypropyl)-2-phenylacetamide (1) (Scheme 1). A previously published study on a similar cascade system starts from aldehyde 2 instead alcohol 1,^53^*i.e.*, an additional step with the ADH enzyme is introduced in the investigated cascade (Scheme 1). The novelty of this research lies not only in different starting material but also in the detailed kinetic analysis aimed at discovering reaction bottlenecks and one-pot synthesis. The investigated reaction is an enzymatically catalyzed cascade reaction, for which the starting material, alcohol 1, was chemically synthesized in our lab. In the first reaction step compound 1 is oxidized catalyzed by alcohol dehydrogenase (ADH) to furnish the corresponding aldehyde, *i.e.*, *N*-(3-oxopropyl)-2-phenylacetamide (2), which is concomitantly consumed in sequential aldol additions with two equivalents of acetaldehyde catalyzed by 2-deoxy-d-ribose-5-phosphate aldolase (DERA) (Scheme 1A). Obviously, the multi-step scheme is complex because an additional cofactor regeneration system is needed. The NADH oxidase (NOX) was selected for this purpose because it has a high driving force. In addition, the selected NOX produces water during the regeneration of the coenzyme NAD^+^, in contrast to hydrogen peroxide, which is produced by the action of some other NADH oxidases.^54^

The aim of this work was to investigate the possibility of using *N*-(3-hydroxypropyl)-2-phenylacetamide (1) as a starting substrate for the synthesis of precursor of the chiral statin side chains in a one-pot enzymatic cascade reaction (Scheme 1). To understand the effects in the reactor and define the bottlenecks in the process, a mathematical modeling approach was applied in this work, meaning that the kinetics of all reactions in this complex cascade were determined. A preliminary value addition evaluation was also performed to compare the approach used in this work (a cascade starting from alcohol 1) with the synthesis of lactol 5 using only aldol addition.^53^ The developed mathematical model was used to describe a more general, physical picture of the process and does not reflect the mechanism of the reaction, which is very complex, especially in DERA-catalyzed aldol condensation, which proceeds in two sequential steps. After the first addition, DERA uses the formed product as an acceptor for the second aldol addition and this mechanism is well documented in the literature.^55,56^ It was not possible to measure the concentration of the product of this first addition and, therefore, our kinetic model does not depict the reaction mechanism completely. This is very common in reaction engineering where simplification is done, under the assumption that the model is valid and describes the behavior of the system well, which is usually quantified by the statistical output.

### Experimental part

### Chemicals

Acetonitrile, acetaldehyde 4, alcohol dehydrogenase (ADH) from horse liver, ethanol 3, 3-hydroxybutanal (dimer 7), triethanolamine hydrochloride (TEA HCl) and trifluoroacetic acid (TFA) were purchased from Sigma-Aldrich (Germany). NAD^+^, NADH, methanol, pyridine and *O*-benzylhydroxylamine were purchased from Acros Organics (USA). Reference materials for *N*-(2-((2*R*,4*R*)-4,6-dihydroxytetrahydro-2*H*-pyran-2-yl)ethyl)-2-2-phenylacetamide (phenylacetamide-lactol 5) and 3,5-dihydroxyhexanal (trimer 8) were synthesized and purified at the Technische Universität Darmstadt (Germany). *N*-(3-Hydroxypropyl)-2-phenylacetamide (alcohol 1) and *N*-(3-oxopropyl)-2-phenylacetamide (aldehyde 2) were isolated and purified at the University of Zagreb Faculty of Chemical Engineering and Technology (Croatia) and Ruđer Bošković Institute (Croatia) (Chapters S1 and S2, ESI†). 2-Deoxyribose-5-phosphate aldolase (DERA 062) and NADH oxidase (NOX 009) were provided by Prozomix Ltd (United Kingdom).

### HPLC analysis

The concentrations of reactants and products were determined by high performance liquid chromatography (HPLC) (Shimadzu, Japan) on a Kinetex® column (250 × 4 mm, 5 μm). Prior to analysis, samples were subjected to derivatization. Samples (5 μL) were mixed with *O*-benzylhydroxylamine (25 μL of a stock solution containing: *O*-benzylhydroxylamine (200 mg), pyridine (6.6 mL), methanol (3 mL) and water (0.4 mL)) for 5 min at 25 °C. Subsequently, 250 μL methanol was added per sample and the mixture was centrifuged. The upper layer was used for HPLC analysis at 30 °C, and a flow rate of 1.5 mL min^−1^, using a gradient method. The mobile phase A was a mixture of acetonitrile and water in a ratio of 80 : 20 with the addition of TFA (0.1%), and the mobile phase B was water with the addition of TFA (0.1%). The method used was as follows: gradient of 10–70% B in the first 12 min, 70% of B from 12–16 min and gradient from 70–10% B from 16–18 min. Detection was performed at 215 nm. The retention times for *N*-phenylacetyl-β-alanine (acid 6), alcohol 1, trimer 8, dimer 7, phenylacetamide-lactol 5, acetaldehyde 4 and aldehyde 2 were 5.4, 5.7, 9.3, 10.1, 11.2, 12.5 and 13.0 min, respectively.

### Enzyme kinetics

The initial rate method was used to investigate the kinetics of each reaction in this cascade system (Scheme 1) separately.^37,38^ This method involves measuring the dependence of the activity of each enzyme on the concentration of each compound in the system. Thus, the concentration of one compound is varied at a time while maintaining the concentration of the other components constant. All measurements were done in duplicates.

The reaction kinetics for all enzymes was determined in 100 mM TEA HCl buffer, pH 8.0, at 25 °C. This pH was selected to benefit the oxidation of alcohol 1 in the cascade reaction because oxidation is favored at higher pH. Kinetic measurements for DERA were carried out in a 1 mL batch reactor (plastic Eppendorf 2 mL-tube) on an orbital shaker at 1000 rpm. The kinetics of ADH and NOX were measured spectrophotometrically in a 1 mL cuvette. The details of all experimental conditions are given in the figure legends (ESI Fig. S1–11†).

Kinetics for all enzymes was determined by monitoring the concentration of the products by HPLC using the previously described method or, for ADH and NOX, using a spectrophotometer for following the absorbance of NADH at 340 nm. The method for measuring the influence of oxygen on NOX activity was described previously^57^ and the oxygen concentration was analyzed using an oxygen electrode (FireSting O2, PyroScience GmbH, Aachen, Germany). All kinetic experiments were carried out at substrate conversion lower than 10%, and the maximum volume of all samples never exceeded 10% of the reaction mixture. The changes in product concentration over time in individual reactions or the changes of absorbance over time^58^ were used to calculate the volume and specific activity of the enzymes.

### Batch reactor experiments

Batch reactor experiments of separate reaction steps, that is, oxidation of the alcohol with coenzyme regeneration, aldol addition of acetaldehyde to aldehyde, side reaction of acetaldehyde dimerization and trimerization, and side reaction of aldehyde oxidation to the corresponding acid, were carried out to validate the developed mathematical model. All reactions were carried out in a 1 mL batch reactor on an orbital shaker at 1000 rpm. They were performed in 100 mM TEA HCl buffer, pH 8.0, at 25 °C. All experimental conditions are indicated in the figure legends.

### Fed-batch reactor experiments

The cascade reaction (Scheme 1) was carried out in the fed-batch reactor (ESI, Fig. S12†) with a continuous feed (*q*_V_ = 0.1 μL min^−1^) of acetaldehyde 4. A syringe pump (PHD 4400 Syringe Pump Series, Harvard Apparatus) with high-pressure stainless-steel piston (8 mL, Harvard Apparatus) was used to deliver the acetaldehyde into the reactor. The initial volume of the reaction mixture was 1 mL and the reactor (Eppendorf tube of 2 mL volume) was placed on an orbital shaker at 1000 rpm. The duration of the addition of acetaldehyde 4 varied from 12, 24 to 48 h. The reactions were performed in 100 mM TEA HCl buffer, pH 8.0, at 25 °C.

### Data handling

A mathematical model was established based on the reaction scheme (Scheme 1) and the kinetic data obtained.

From the obtained experimental data sets of specific activity *vs.* concentration, apparent kinetic parameters *V*_m_, *K*_m_, *K*_i_ were estimated. Non-linear regression methods (*i.e.*, simplex and least squares fit) in the software SCIENTIST 2.0 ^59^ were used for that purpose and the same software was used for model simulations. Enzyme operational stability decay rate constants (*k*_d_) were estimated from the batch reactor experiments.

## Results and discussion

The results of the experiments were divided into four sections: (i) Enzyme kinetics; (ii) Mathematical model development, (iii) Mathematical model validation; and (iv) Model-based observations.

### Enzyme kinetics

The kinetics of each reaction in the cascade system (Scheme 1) was investigated separately. All results, figures and kinetic parameters are presented in ESI (Chapter S3, Fig. S1–11 and Tables S1–S9).† ADH-catalyzed oxidation of alcohol 1 was severely inhibited by NADH (Fig. S1C, *K*^NADH^_i1_ = 8.987 μM, Table S1†) and aldehyde 2 (Fig. S1D, *K*^aldehyde^_i1_ = 1.967 mM, Table S1†) but also by acetaldehyde 4 (Fig. S1E, *K*^acetaldehyde^_i1_ = 1.646 mM, Table S1†), which is the substrate of the second step, *i.e.*, the aldol addition catalyzed by DERA. Additionally, ADH showed relatively low affinity towards alcohol 1 as a substrate (Fig. S1A, *K*^alcohol^_m1_ = 51.919 mM, Table S1†). These findings imply that this synthetic process (Scheme 1) must be carried out as a cascade in which all reactions proceed simultaneously to minimize the negative effect of aldehyde 2 on oxidation, *i.e.*, to keep its concentration low due to its immediate consumption in the subsequent aldol addition. Additionally, due to the inhibition by acetaldehyde 4, this compound should be fed into the reactor to keep its concentration as low as possible. With efficient coenzyme regeneration, the NADH concentration can be kept to a minimum, so that the inhibitory effect of NADH is minimized.

On the other hand, ADH shows a higher *K*_m_ towards aldehyde 2 in the reduction reaction (Fig. S2A, *K*^aldehyde^_m2_ = 1.981 mM, Table S1†), than towards alcohol 1 in the oxidation reaction (*K*^alcohol^_m1_ = 51.919 mM, Table S1†). In addition, the maximum reaction rate of the reduction is higher than the maximum reaction rate of the oxidation (*V*_m2_ = 0.626 U mg^−1^ and *V*_m1_ = 0.109 U mg^−1^, respectively, Table S1†). The reduction reaction is significantly inhibited by NAD^+^ (Fig. S2C, 
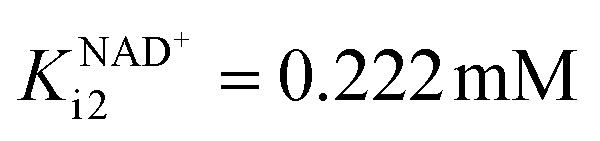
, Table S1†) and ethanol 3 (Fig. S2D, *K*^ethanol^_i2_ = 1.493 mM, Table S1†) and less inhibited by alcohol 1 (Fig. S2E, *K*^alcohol^_i2_ = 81.240 mM, Table S1†). As far as NAD^+^ inhibition is concerned, it is beneficial that the reduction reaction is inhibited with NAD^+^. When the coenzyme is efficiently regenerated, the NAD^+^ concentration is at its highest and ensures the greatest possible inhibition, which helps to shift the oxidoreduction equilibrium in favor of product formation.

In this cascade reaction, NOX was used for NAD^+^ regeneration. The kinetics of this enzyme (Fig. S3†) shows that it is very efficient and has a high affinity (Table S2†) towards its substrates, NADH (Fig. S3A,†*K*^NADH^_m3_ = 0.075 mM, Table S2†) and oxygen (Fig. S3B, 
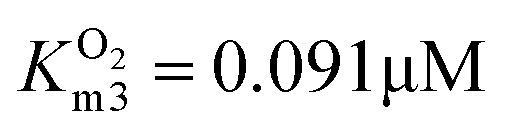
, Table S2†). It was assumed that the concentration of oxygen in the reaction mixture is at all times sufficient to ensure maximal enzyme activity due to the high affinity of the enzyme towards oxygen (
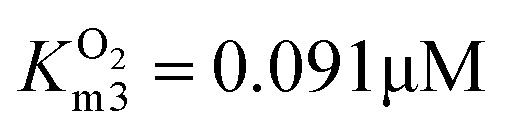
, Table S2†). Hence, its mass balance and its concentration were not included in the equation to keep the model as simple as possible. It was shown that NOX is inhibited by NAD^+^ (Fig. S3C, 
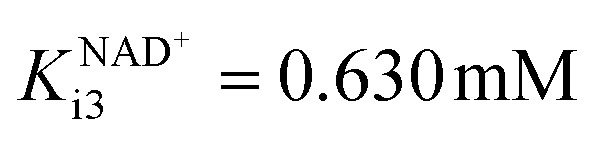
, Table S2†), ethanol 3 (Fig. S3D, *K*^ethanol^_i3_ = 0.678 mM, Table S2†), acetaldehyde 4 (Fig. S3E, *K*^acetaldehyde^_i3_ = 77.326 mM, Table S2†), alcohol 1 (Fig. S3F, *K*^alcohol^_i3_ = 42.265 mM, Table S2†) and aldehyde 2 (Fig. S3G, *K*^aldehyde^_i3_ = 3.361 mM, Table S2†). All these inhibitions can negatively affect the efficiency of coenzyme regeneration depending on the concentrations of the inhibitors in the reactor.

ADH also catalyzes reversibly the reduction of the acetaldehyde 4 present (Scheme 1). Even though this reaction could be an alternative coenzyme regeneration system, the kinetic parameters show (Table S3†) that acetaldehyde 4 reduction is strongly inhibited by NAD^+^ (Fig. S4D,†
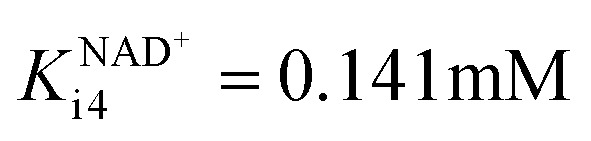
) and alcohol 1 (Fig. S4E,†*K*^alcohol^_i4_ = 0.041 mM), which indicates low efficiency of this reaction as a coenzyme regeneration system. The reverse reaction, ethanol 3 oxidation, is severely inhibited by acetaldehyde 4 (Fig. S5C, *K*^acetaldehyde^_i5_ = 3.858 μM, Table S3†) and NADH (Fig. S5D, *K*^NADH^_i5_ = 2.911 μM, Table S3†).

In the aldol addition catalyzed by DERA, two equivalents of acetaldehyde 4 react with aldehyde 2 to form the major product of this reaction, phenylacetamide-lactol 5. This reaction is moderately inhibited by acetaldehyde 4 (Fig. S6B, *K*^acetaldehyde^_i6_ = 723.802 mM, Table S4†). There are some additional inhibitions by alcohol 1 (Fig. S6C, *K*^alcohol^_i6_ = 68.103 mM, Table S4†), ethanol 3 (Fig. S6D, *K*^ethanol^_i6_ = 4.872 mM, Table S4†) and NAD^+^ (Fig. S6E, 
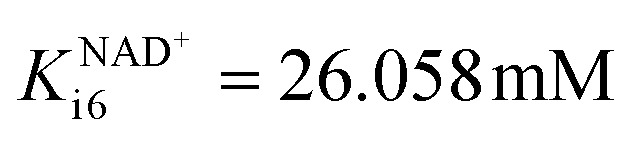
, Table S4†), which are less significant, and by trimer 8 (*i.e.* 3,5-dihydroxyhexanal, *K*^trimer^_i6_ = 0.641 mM, Table S4†), which is more significant. The inhibition by trimer 8 was estimated using a batch reactor experiment (Fig. 4).

In addition to the aldol addition, aldehyde 2 can also be oxidized to acid 6, which is catalyzed by all three enzymes present in the cascade (Fig. S7–S9†). However, all three reactions are very slow as shown by the estimated maximum reaction rates (*V*_m7_ = 0.006 U mg^−1^, Table S5, *V*_m8_ = 1.416 × 10^−3^ U mg^−1^, Table S6, *V*_m9_ = 0.0078 U mg^−1^, Table S7†) and should not generate high concentrations of this by-product.

The main challenge in this cascade reaction is the competing side reactions of acetaldehyde dimerization (*i.e.*, product 7) (Fig. S10†) and trimerization (*i.e.*, product 8) (Fig. S11†). These reactions are catalyzed by DERA and have 1.4- and 2-fold higher maximum reaction rates (*V*_m10_ = 0.248 U mg^−1^, Table S8, *V*_m11_ = 0.332 U mg^−1^, Table S9†), respectively, than the desired aldol addition (*V*_m6_ = 0.174 U mg^−1^, Table S4†). The major advantage is that DERA has a higher apparent affinity towards acetaldehyde 4 in the aldol addition (*K*^acetaldehyde^_m6_ = 1.927 mM, Table S4†) than in dimerization (*K*^acetaldehyde^_m10_ = 39.009 mM, Table S8†) and trimerization reactions (*K*^acetaldehyde^_m11_ = 69.960 mM, Table S9†). To use this advantage in favor of the formation of the wanted product, and to avoid the *V*_m_ ranges for the formation of the by-products, the conditions for carrying out the cascade reaction must be carefully selected. From the estimated values of the Michaelis constants it can be concluded that the acetaldehyde 4 concentration needs to be kept low in the reactor to reduce the formation of the by-products. Indeed, from the calculations/simulations of the reaction rates, it follows that at an acetaldehyde 4 concentration of 10 mM in the reactor, the reaction rate of aldol addition (Fig. S6BI†) should reach the maximum value, while the rate of dimerization (Fig. S10†) and trimerization (Fig. S11A†) will be significantly reduced, but still present.

### Mathematical model development

ADH catalyzes the oxidoreduction (*r*_1_, *r*_2_), *i.e.*, the oxidation of alcohol 1 (*r*_1_) and the reduction of aldehyde 2 (*r*_2_). The oxidation of alcohol 1 (*r*_1_) was described by the double-substrate Michaelis–Menten equation with included competitive inhibition by aldehyde 2, acetaldehyde 4 and NADH (eqn (1), Table 1). Double-substrate Michaelis–Menten equation with included substrate (aldehyde 2) inhibition and competitive inhibition by alcohol 1, ethanol 3 and NAD^+^ (eqn (2), Table 1) was used to describe the reduction of the aldehyde 2 (*r*_2_). NOX-catalyzed coenzyme regeneration (*r*_3_) was described by the Michaelis–Menten equation with competitive inhibition by alcohol 1, aldehyde 2, acetaldehyde 4 and ethanol 3 and uncompetitive inhibition by NAD^+^^57,60^ (eqn (3), Table 1). ADH also catalyzes the reduction of acetaldehyde 4 to ethanol 3 (*r*_4_) and the oxidation of ethanol 3 to acetaldehyde 4 (*r*_5_), which can also be considered a coenzyme regeneration system and an additional way of acetaldehyde consumption. Double-substrate Michaelis–Menten equation with included substrate (acetaldehyde 4) inhibition and competitive inhibition by alcohol 1, ethanol 3 and NAD^+^ (eqn (4), Table 1) was used to describe the reduction of acetaldehyde 4 (*r*_4_). The reverse reaction, the oxidation of ethanol 3 (*r*_5_), was also described by double-substrate Michaelis–Menten equation with included substrate (ethanol 3) inhibition and competitive inhibition by aldehyde 2, acetaldehyde 4 and NADH (eqn (5), Table 1). The main product, phenylacetamide-lactol 5, is synthesized in a reaction catalyzed by DERA (*r*_6_) which was described by three-substrate Michaelis–Menten equation with included substrate (acetaldehyde 4) inhibition and competitive inhibition by the alcohol 1, ethanol 3 and NAD^+^ (eqn (6), Table 1). Although the aldol addition is a consecutive two-fold addition, it was not possible to measure the concentration of the intermediate which is necessary to calculate the reaction rate of an investigated reaction. Therefore, the presented mathematical model does not reflect the mechanism of the reaction but describes a more general, physical picture of the process. The reverse reaction, a double retro-aldol fragmentation, was omitted as it was found to be insignificant.

**Table 1:** Mathematical model for the cascade reaction: kinetic equations

Kinetic equations	
**ADH-catalyzed oxidoreduction**	
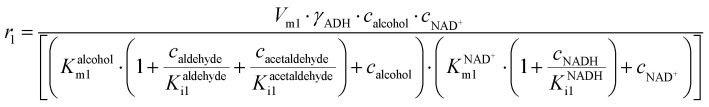	(1)
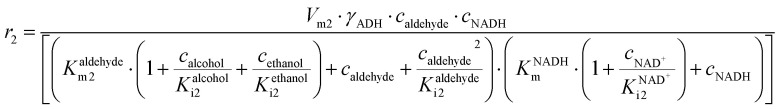	(2)

**NOX-catalyzed coenzyme regeneration**	
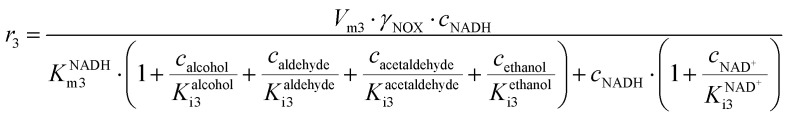	(3)

**ADH-catalyzed coenzyme regeneration (acetaldehyde reduction)**	
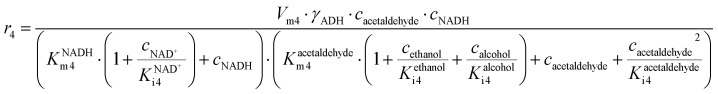	(4)
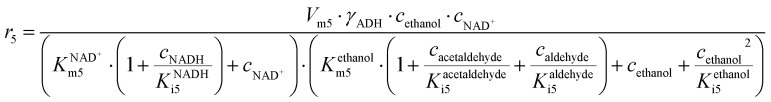	(5)

**DERA-catalyzed aldol addition of aldehyde and acetaldehyde**	
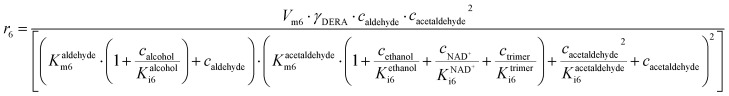	(6)

**ADH-catalyzed aldehyde oxidation**	
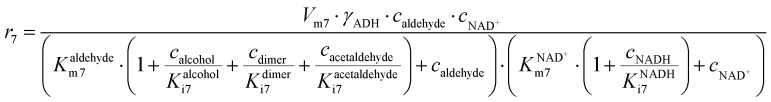	(7)

**DERA-catalyzed aldehyde oxidation**	
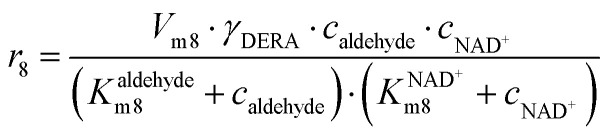	(8)

**NOX-catalyzed aldehyde oxidation**	
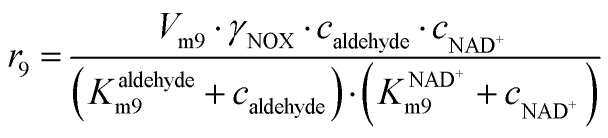	(9)

**DERA-catalyzed dimerization of acetaldehyde**	
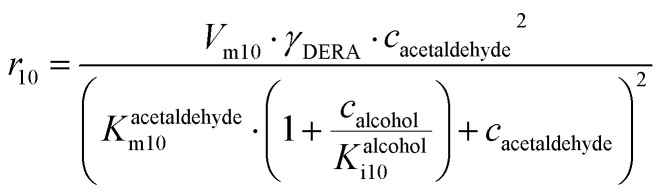	(10)

**DERA-catalyzed trimerization of acetaldehyde**	
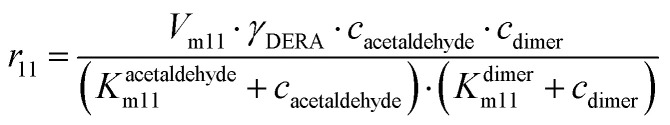	(11)

**Operational stability decay rate**	
*V*_m_ = *V*_m0_ e^−*k*_d_*t*^	(12)

Apart from the main product, three undesirable by-products are formed in this cascade system: acid 6, dimer of acetaldehyde 7 and trimer of acetaldehyde 8. ADH (*r*_7_), DERA (*r*_8_) and NOX (*r*_9_) catalyze the aldehyde oxidation in which the acid 6 is formed. Aldehyde 2 oxidation catalyzed by ADH (*r*_7_) was described by double-substrate Michaelis–Menten equation with included competitive inhibition by the alcohol 1, dimer 7 and acetaldehyde 4 (eqn (7), Table 1). The same reaction catalyzed by DERA (*r*_8_) and NOX (*r*_9_) was described by double-substrate Michaelis–Menten equation (eqn (8) and (9), respectively, Table 1). DERA also catalyzes the formation of acetaldehyde dimer 7 (*r*_10_) and trimer 8 (*r*_11_). The dimerization of acetaldehyde 4 was described by double-substrate (acetaldehyde 4) Michaelis–Menten equation (eqn (10), Table 1) and the trimerization by double-substrate Michaelis–Menten equation with alcohol 1 inhibition (eqn (11), Table 1). The possible oxidation of acetaldehyde 4 to acetic acid was considered insignificant due to the excess of acetaldehyde 4 in the system and was not further investigated. The decay of operational stability of the enzymes was described by the kinetics of the first order (eqn (12), Table 1).

The mass balance equations for all components of the reaction in the batch (eqn (13)–(22)) and the fed-batch reactor (eqn (23)–(35)) are presented in Table 2. Eqn (36) (Table 2) describes the change in the reactor volume due to the feed of acetaldehyde 4.

**Table 2:** Mathematical model for the cascade reaction: mass balance equations

Mass balances in the batch reactor	
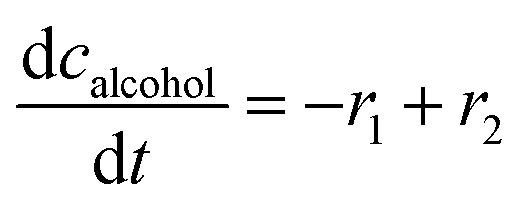	(13)
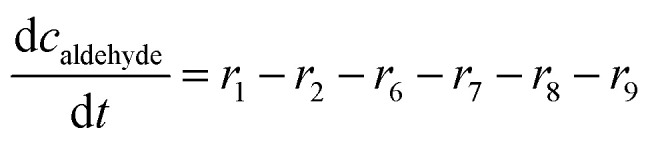	(14)
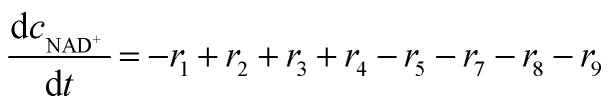	(15)
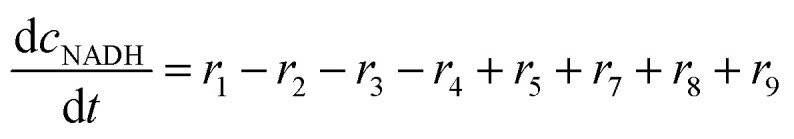	(16)
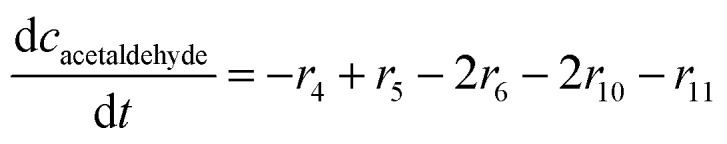	(17)
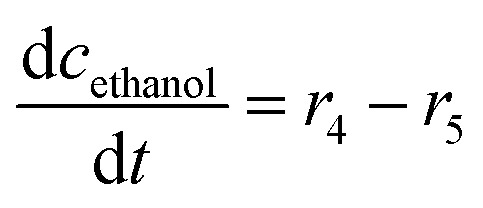	(18)
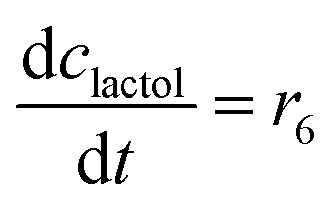	(19)
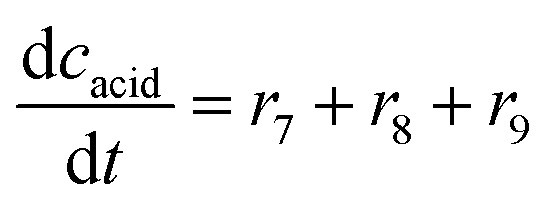	(20)
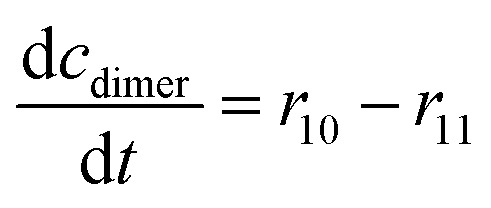	(21)
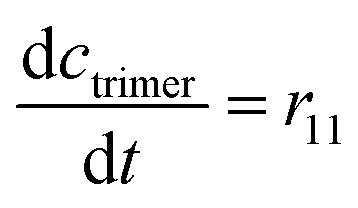	(22)

**Mass balances in the fed batch reactor**	
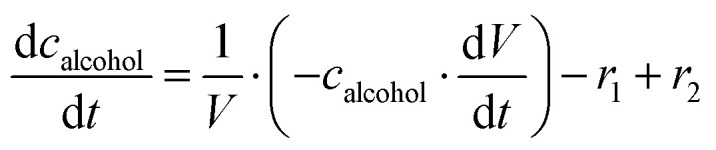	(23)
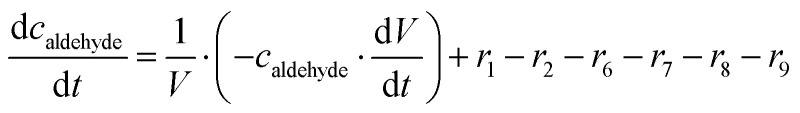	(24)
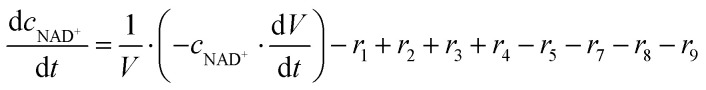	(25)
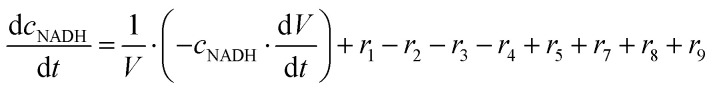	(26)
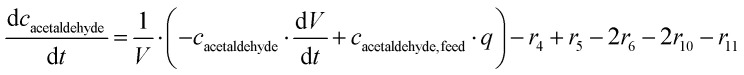	(27)
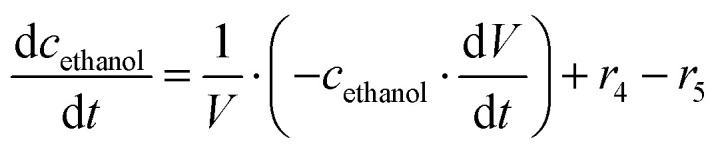	(28)
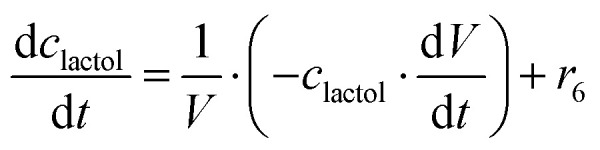	(29)
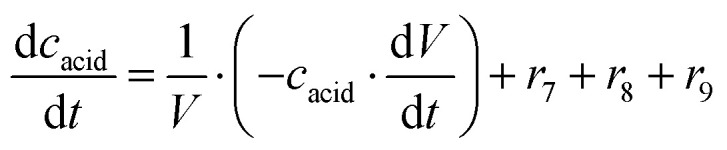	(30)
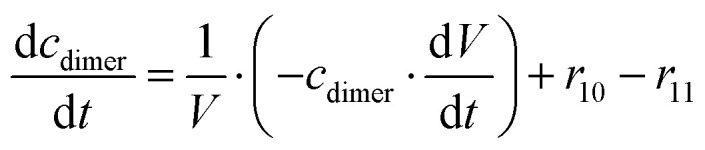	(31)
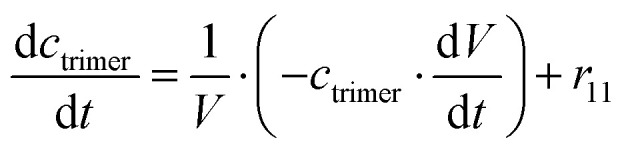	(32)
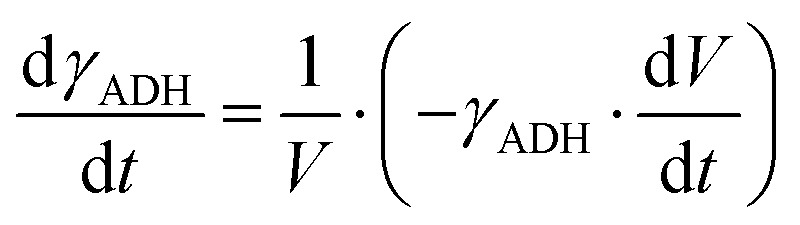	(33)
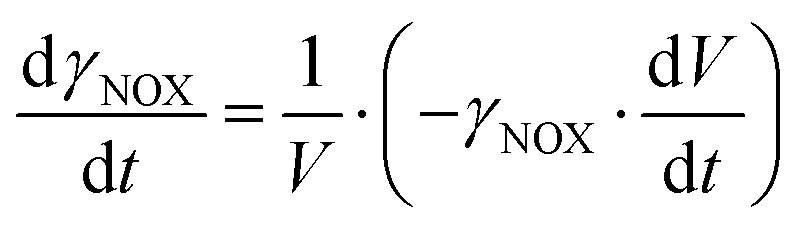	(34)
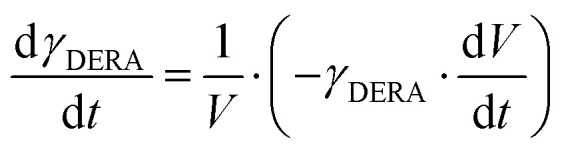	(35)
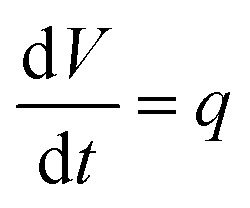	(36)

### Model validation

Based on the enzyme kinetic measurements (ESI, Chapter S3†), the corresponding estimated kinetic parameters (Tables S1–S9†), as well as the reaction sequence (Scheme 1), the kinetic model of the cascade was developed (Table 1). To validate the estimated kinetic parameters and the developed mathematical model, specific reactions were carried out separately in the batch reactor and are shown and described in the following paragraphs.

**Fig. 1:**
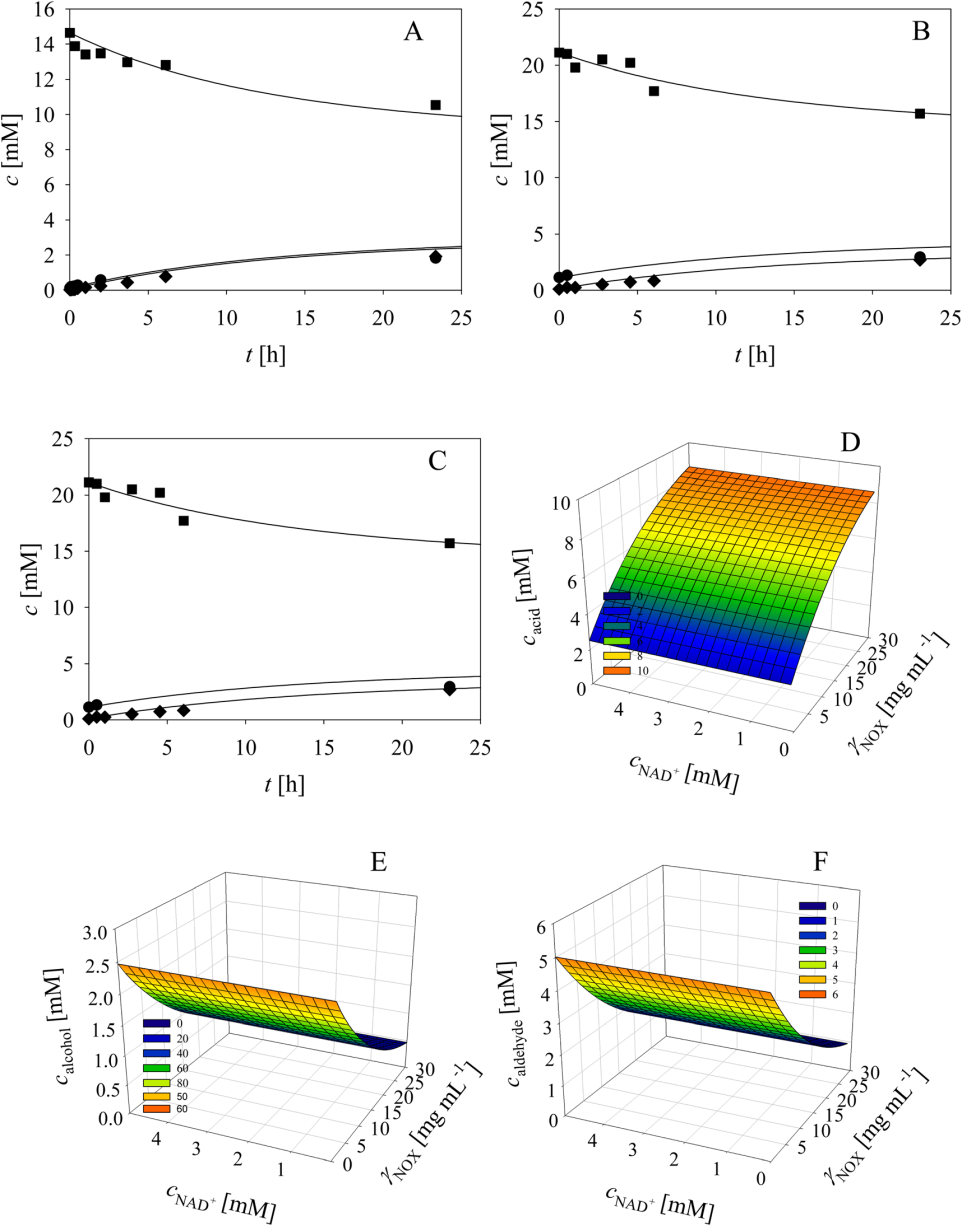
Side reaction of aldehyde oxidation catalyzed by ADH – formation of acid (100 mM TEA HCl pH 8.0, *γ*_ADH_ = 1 mg mL^−1^, (A) *c*_aldehyde_ = 14.2 mM, *c*_NAD^+^_ = 8.99 mM, (B) *c*_aldehyde_ = 21.12 mM, *c*_NAD^+^_ = 24.99 mM, (C) *c*_aldehyde_ = 33.46 mM, *c*_NAD^+^_ = 49.98 mM). Legend: black circles – alcohol, black squares – aldehyde, black diamonds – acid, line – model. Influence of NAD^+^ and NOX concentration on the concentration of (D) acid, (E) alcohol and (F) aldehyde in the reaction of aldehyde oxidation catalyzed by ADH with coenzyme regeneration catalyzed by NOX and alcohol oxidation catalyzed by ADH (*c*_aldehyde_ = 10 mM, *γ*_ADH_ = 1 mg mL^−1^).

As evident from the previous section, the reactions and side-reactions catalyzed by the three enzymes are complex and interdependent (Scheme 1). Thus, it was intended to carry out experiments in which the interdependent reactions and side-reactions were minimized to eliminate the side effects of the different enzymes. To this end, we began with the side reactions in which acid 6 is formed. Three reactions of aldehyde 2 oxidation catalyzed by ADH (Scheme 1, *r*_7_) at different initial concentrations of aldehyde 2 and NAD^+^ were carried out (Fig. 1) to validate the model (eqn (1), (2), (7) and (12), Table 1, eqn (13)–(16) and (20), Table 2), as well as to estimate the operational stability decay rate constant of ADH (Table S10†). It should be noted that some parts in the mass balance equations (eqn (13)–(16), Table 2) are zero and only *r*_1_, *r*_2_, *r*_7_ are positive in this case. The operational stability decay rate constant of ADH (Table S10†) was estimated from the data of the experiment presented in Fig. 1A and used for the simulation of all other experiments, including the oxidation of alcohol 1 with coenzyme regeneration and cascade reaction presented later. The results (Fig. 1) show that low concentration of the acid 6 is formed during 24 h, as expected based on the measured kinetics. Additionally, alcohol 1 is formed in this reaction, which was to be expected since ADH also catalyzes the reduction of aldehyde 2. The mathematical model (eqn (1), (2), (7) and (12), Table 1, eqn (13)–(16) and eqn (20), Table 2) described all reactions well, which can be substantiated by the statistical output of the SCIENTIST software (Table S11†). A higher acid 6 concentration is expected with included coenzyme regeneration. Simulation of the influence of NAD^+^ and NOX concentration (eqn (1)–(3), (7), (9) and (12), Table 1, eqn (13)–(16) and (20), Table 2) on acid 6 concentration (Fig. 1D) shows that NAD^+^ concentration has no influence on the final acid 6 concentration in the investigated range. On the other hand, the NOX concentration has a significant influence, *i.e.*, higher acid 6 concentration is formed with increasing NOX concentration reaching an almost quantitative conversion at a NOX concentration of 30 mg mL^−1^ (Fig. 1D). This is partly due to the presence of a coenzyme regeneration system but probably also to the fact that NOX itself catalyzes aldehyde oxidation to acid to a certain extent (ESI, Chapter S3.9†).

**Fig. 2:**
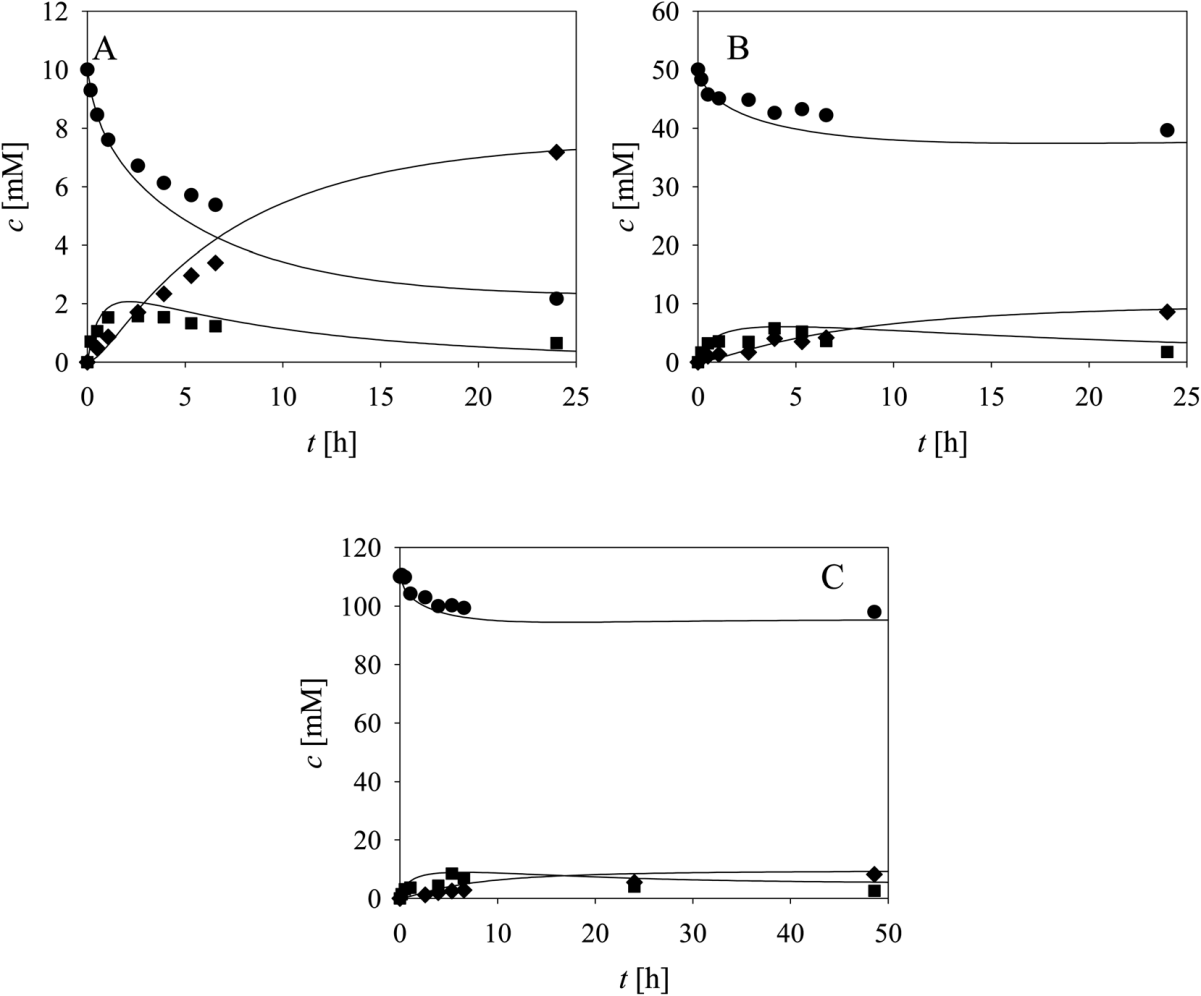
Alcohol oxidation catalyzed by ADH with coenzyme regeneration catalyzed by NOX (100 mM TEA HCl pH 8.0, *γ*_ADH_ = 10 mg mL^−1^, *γ*_NOX_ = 10 mg mL^−1^, *c*_NAD^+^_ = 1.02 mM, (A) *c*_alcohol_ = 13.01 mM, (B) *c*_alcohol_ = 50.20 mM, (C) *c*_alcohol_ = 109.80 mM). Legend: black circles – alcohol, black squares – aldehyde, black diamonds – acid, line – model.

In the next validation stage, the focus was placed on the first reaction step, *i.e.*, the oxidation of alcohol 1 to aldehyde 2, with NAD^+^ regeneration (Scheme 1, *r*_1_, *r*_3_) catalyzed by NOX (Fig. 2). Both the oxidation reaction and the formation of acid 6 (Scheme 1, *r*_7_, *r*_9_), (ESI, Chapters S3.7 and S3.9†) were included in the model. The operational stability decay rate constant of NOX (Table S10, ESI†) was estimated from the experiment presented in Fig. 2A. Even though this constant was introduced into the model (eqn (1)–(3), (7), (9) and (12), Table 1, eqn (13)–(16) and (20), Table 2), the model initially showed that a lower acid concentration was formed than in the experiment. Therefore, the maximum reaction rate of aldehyde 2 oxidation catalyzed by ADH was re-estimated (Table S5,†*V*_m7_ value in the brackets) based on the experiment presented in Fig. 2A, since the same enzyme, ADH, catalyzes both alcohol 1 and aldehyde 2 oxidation. The new *V*_m7_ value was 2.7-fold higher (*V*_m7_ = 0.016 U mg^−1^, Table S5†) than the value estimated from the independent kinetic experiments (*V*_m7_ = 0.006 U mg^−1^, Table S5†). This could be explained by the absence of the enzyme–substrate complex formation step in aldehyde 2 oxidation. The aldehyde 2 is already at the active site and is ready to be further oxidized to acid 6 meaning there is no diffusion of the aldehyde into the reaction mixture and back. This new *V*_m7_ value was used for all subsequent simulations presented in this work. Two further alcohol 1 oxidation experiments with coenzyme regeneration were carried out (Fig. 2B and C). These experiments were described well by the mathematical model (eqn (1)–(3), (7), (9) and (12), Table 1, eqn (13)–(16) and (20), Table 2). This conclusion was substantiated by the statistical data (Table S11†). In the experiments presented in Fig. 2, the concentration of aldehyde 2 is low due to its further oxidation to acid 6. These results show the necessity to carry out all reactions of the cascade simultaneously to shift the equilibrium towards the formation of the target product 5.

**Fig. 3:**
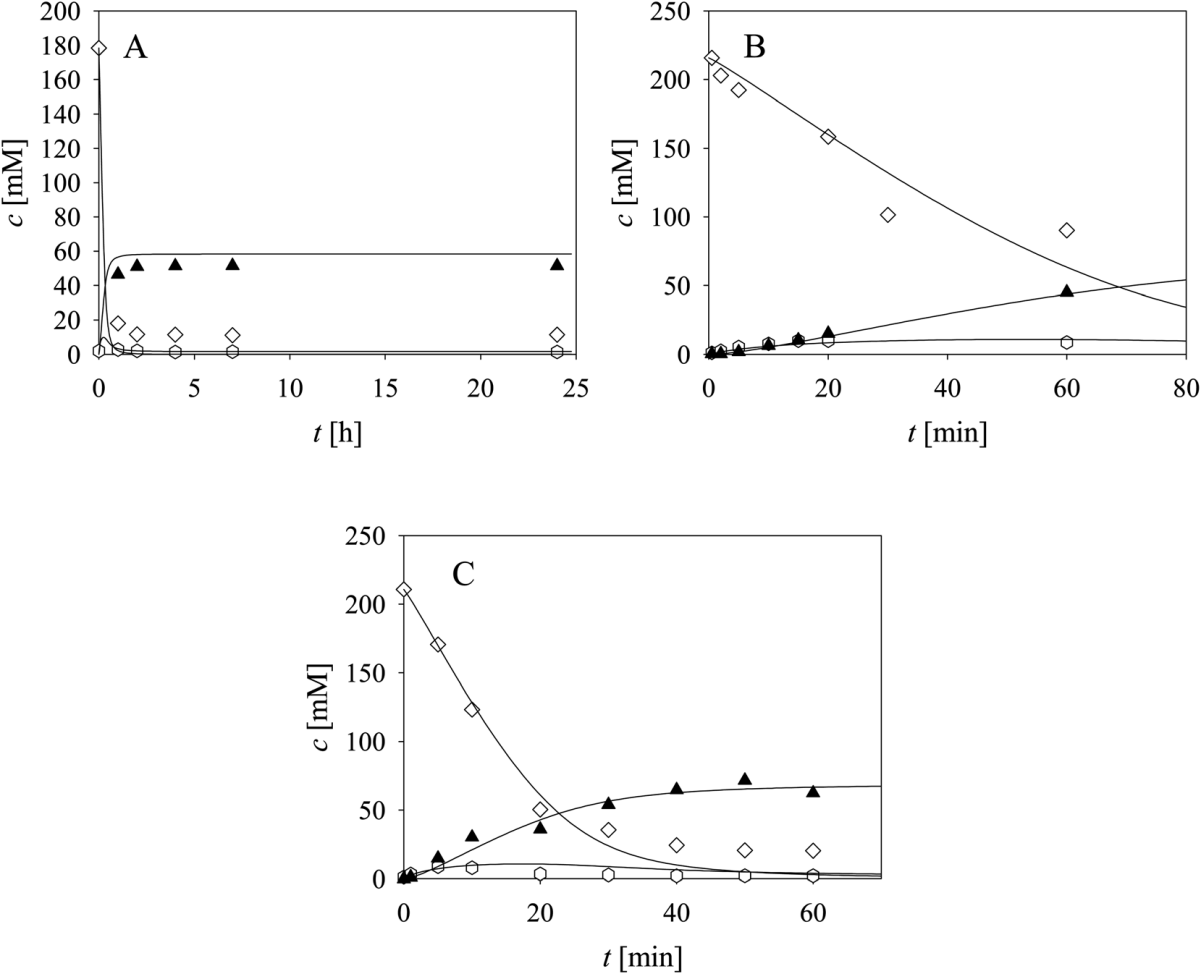
Side reaction of acetaldehyde dimerization and trimerization catalyzed by DERA (100 mM TEA HCl pH 8.0, 25 °C, *V*_r_ = 1 mL, 1000 rpm, (A) *γ*_DERA_ = 20.2 mg mL^−1^, *c*_acetaldehyde_ = 178.34 mM, (B) *γ*_DERA_ = 6.3 mg mL^−1^, *c*_acetaldehyde_ = 215.85 mM, (C) *γ*_DERA_ = 20.0 mg mL^−1^, *c*_acetaldehyde_ = 210.78 mM). Legend: white diamonds – acetaldehyde, white hexagons – dimer, black triangles – trimer, line – model.

Two additional side reactions occur in this system. DERA catalyzes acetaldehyde dimerization and trimerization, reactions in which dimer 7 (Scheme 1, *r*_10_), and trimer 8 (Scheme 1, *r*_11_), are formed. These reactions are enzyme-catalyzed since no products were formed after 24 h without DERA (data not presented). To validate the model developed for these two side reactions (eqn (10)–(12), Table 1, eqn (17), (21) and (22), Table 2), three reactions were carried out. The first reaction was monitored during 24 h (Fig. 3A) and the next two reactions (Fig. 3B and C), at different DERA concentrations, *i.e.*, 6.3 and 20 mg mL^−1^, were followed during 1 h since they were very fast. The model (eqn (10)–(12), Table 1, eqn (17), (21) and (22), Table 2) described all three experiments well (Fig. 3), which was substantiated by the statistical data (Table S11†). The reactions of acetaldehyde dimerization and trimerization are fast. Therefore, it is important to find the right conditions to minimize them, *e.g.*, by minimizing the concentration of acetaldehyde 4 in the reactor by feeding it into the reactor.

**Fig. 4:**
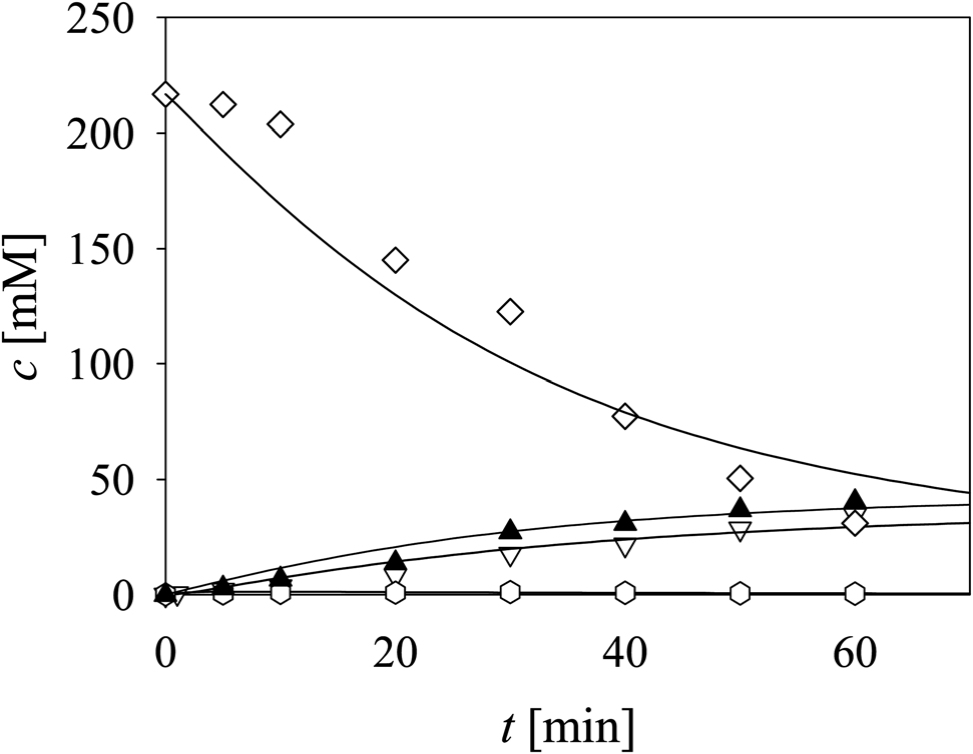
Aldol addition of acetaldehyde to aldehyde catalyzed by DERA (100 mM TEA HCl pH 8.0, 25 °C, *V*_r_ = 1 mL, 1000 rpm, *γ*_DERA_ = 20.4 mg mL^−1^, *c*_acetaldehyde_ = 216.52 mM, *c*_aldehyde_ = 100.94 mM). Legend: white triangles – lactol, white diamonds – acetaldehyde, white hexagons – dimer, black triangles – trimer, line – model.

The aldol addition of two equivalents of acetaldehyde 4 to aldehyde 2 was the last part of the model to be analyzed separately (Scheme 1, *r*_6_). Initially, the model showed some discrepancies with the experimental data, thus, it was assumed that additional interdependencies exist in the cascade reaction that were not investigated in the first round of kinetic measurements. Therefore, two additional parameters, *i.e.*, the inhibition by alcohol 1 in acetaldehyde dimerization (*r*_10,_ Table S8,†*K*^alcohol^_i10_) and inhibition by trimer 8 in the aldol addition (*r*_6_, Table S4,†*K*^trimer^_i6_), estimated from the experiment presented in Fig. 4, were included in the model (eqn (6) and (10)–(12), Table 1, eqn (17), (19), (21) and (22), Table 2). Additionally, the operational stability decay rate constant for DERA was estimated (Table S10†) from the experiment presented in Fig. 4. With these additional parameters, the model described the data well, substantiated by the statistical analysis (Table S11†). The results show that a higher concentration of trimer 8 (40.2 mM) was obtained than the target phenylacetamide-lactol 5 (34.4 mM) under the conditions tested. This was expected considering that high acetaldehyde 4 concentration is present in the system enabling the maximum reaction rates of by-product formation. For this reason, the cascade reaction experiments were carried out in a fed-batch reactor, feeding it with acetaldehyde 4 on the assumption that this would slow down the formation of the acetaldehyde trimer 8.

**Fig. 5:**
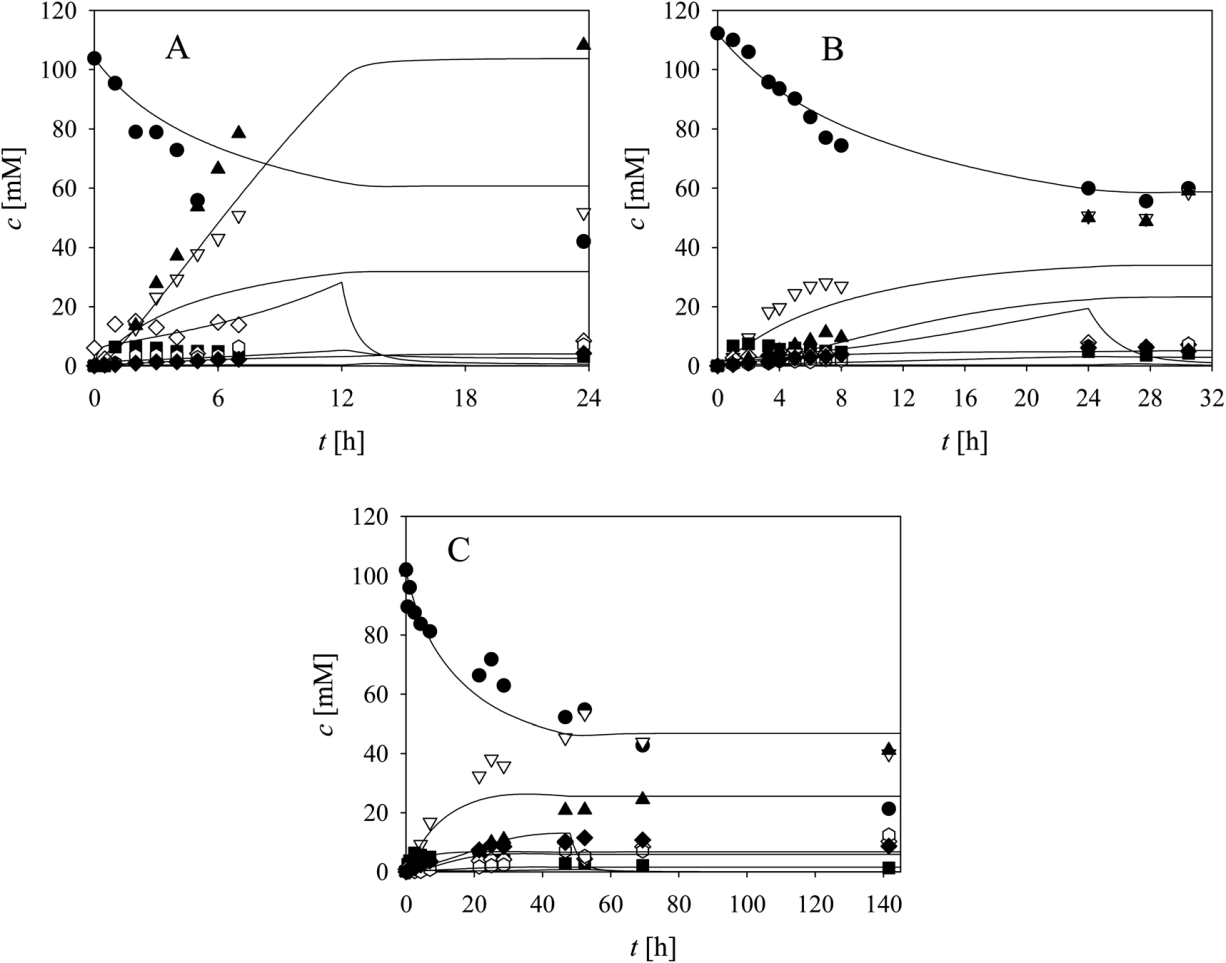
Cascade reaction in the fed-batch reactor (*q*_feed_ = 0.1 μL min^−1^, 100 mM TEA HCl pH 8.0, *V*_r_ = 1 mL, 1000 rpm) with acetaldehyde feed during (A) 12 hours (*c*_acetaldehyde,feed_ = 7 M, *c*_alcohol_ = 103.72 mM, *c*_NAD^+^_ = 5.27 mM, *γ*_ADH_ = 10.7 mg mL^−1^, *γ*_NOX_ = 1.2 mg mL^−1^, *γ*_DERA_ = 20.3 mg mL^−1^), (B) 24 hours (*c*_acetaldehyde,feed_ = 2 M, *c*_alcohol_ = 112.27 mM, *c*_NAD^+^_ = 4.96 mM, *γ*_ADH_ = 10 mg mL^−1^, *γ*_NOX_ = 1 mg mL^−1^, *γ*_DERA_ = 20.1 mg mL^−1^), (C) 48 hours (*c*_acetaldehyde,feed_ = 1 M, *c*_alcohol_ = 100 mM, *c*_NAD^+^_ = 4.97 mM, *γ*_ADH_ = 10 mg mL^−1^, *γ*_NOX_ = 1.1 mg mL^−1^, *γ*_DERA_ = 20.2 mg mL^−1^). Legend: black circles – alcohol, black squares – aldehyde, white triangles – lactol, white diamonds – acetaldehyde, white hexagons – dimer, black triangles – trimer, black diamonds – acid, line – model.

Three cascade reaction experiments carried out in the fed-batch reactor are presented in Fig. 5. The mathematical model described the data well when the newly estimated *V*_m7_ was used for the simulation (Table S5,†*V*_m7_ value in the brackets). The literature^61–64^ and our previous research^37^ have shown that in complex systems, such as this cascade, enzymes work closely together and the interaction between aldolases and dehydrogenases,^65,66^ as well as their proximity, improve the reaction outcome due to substrate channeling.

### Model-based observations

The developed and validated mathematical model was applied to navigate the designed space and evaluate the effect of the process variables on the outcome with special focus on the concentration of the target product and unwanted by-products. The observations are based on the respective enzyme preparation used, which were standardized within this work and originated from one production batch. All simulations were performed for the fed-batch reactor with continuous addition of acetaldehyde 4 during 12 h. The variables investigated were the concentration of the enzymes, ADH, NOX and DERA, the acetaldehyde 4 feed flow rate, the concentration of the acetaldehyde 4 stock solution and the concentration of the coenzyme NAD^+^.

**Fig. 6:**
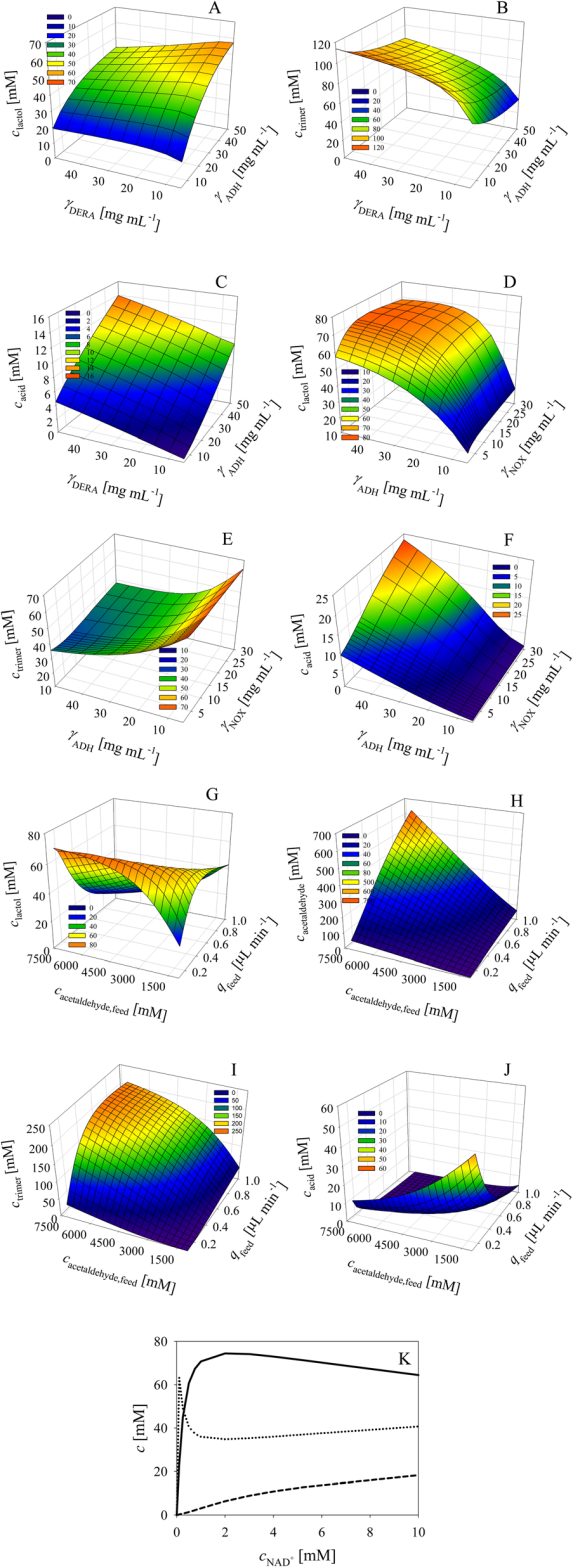
Influence of DERA and ADH concentration on (A) lactol, (B) trimer and (C) acid concentration in the cascade reaction in the fed-batch reactor with acetaldehyde feed during 12 hours (*c*_acetaldehyde,feed_ = 7 M, *q*_feed_ = 0.1 μL min^−1^, *c*_alcohol_ = 100 mM, *c*_NAD^+^_ = 5 mM, *γ*_NOX_ = 5 mg mL^−1^). Influence of ADH and NOX concentration on (D) lactol, (E) trimer and (F) acid concentration in the cascade reaction in the fed-batch reactor with acetaldehyde feed during 12 hours (*c*_acetaldehyde,feed_ = 7 M, *q*_feed_ = 0.1 μL min^−1^, *c*_alcohol_ = 100 mM, *c*_NAD^+^_ = 5 mM, *γ*_DERA_ = 5 mg mL^−1^). Influence of the flowrate and the concentration of acetaldehyde in the feed on (G) lactol, (H) acetaldehyde, (I) trimer and (J) acid concentration in the cascade reaction in the fed-batch reactor with acetaldehyde feed during 12 hours (*c*_alcohol_ = 100 mM, *c*_NAD^+^_ = 5 mM, *γ*_ADH_ = 10 mg mL^−1^, *γ*_NOX_ = 5 mg mL^−1^, *γ*_DERA_ = 5 mg mL^−1^). (K) Influence of NAD^+^ concentration on lactol, trimer and acid concentration in the cascade reaction in the fed-batch reactor with acetaldehyde feed during 12 hours (*c*_acetaldehyde,feed_ = 7 M, *q*_feed_ = 0.1 μL min^−1^, *c*_alcohol_ = 100 mM, *γ*_ADH_ = 10 mg mL^−1^, *γ*_NOX_ = 5 mg mL^−1^, *γ*_DERA_ = 5 mg mL^−1^). Legend: solid line – lactol concentration, dotted line – trimer concentration, dashed line – acid concentration.

The effect of DERA and ADH concentration on the phenylacetamide-lactol 5 final concentration indicates that ADH must be increased up to the maximum value of the investigated range, *i.e.*, 50 mg mL^−1^ (Fig. 6A). At the same time, the concentration of DERA must be decreased to 5 mg mL^−1^. These conditions will ensure a minimum amount of trimer 8 (Fig. 6B). The effect of DERA and ADH concentration on the concentration of acid 6 is presented in Fig. 6C. Under the conditions selected to obtain a maximum amount of phenylacetamide-lactol 5, *i.e.*, 50 mg per mL ADH and 5 mg per mL DERA, the average concentration of acid 6 is not negligible (9.2 mM), but tolerable given the amount of phenylacetamide-lactol 5 (57.3 mM).

In the next round of simulations, the effect of ADH and NOX concentration on the process outcome was considered at a fixed concentration of DERA (5 mg mL^−1^). The results (Fig. 6D) show that the concentration of phenylacetamide-lactol 5 can be increased up to 70 mM (from a maximum of 100 mM) by increasing the NOX concentration from the initial 5 mg mL^−1^ up to 10 mg mL^−1^ and with the ADH concentration to 50 mg mL^−1^. Under these conditions, the concentration of trimer 8 is the lowest (approx. 33 mM, Fig. 6E) and the concentration of acid 6 is acceptable (approx. 12 mM, Fig. 6F).

Additionally, the effect of acetaldehyde feed flow rate and stock solution concentration on the process outcome was evaluated at the best enzyme concentrations previously found, 50 mg per mL ADH, 10 mg per mL NOX and 5 mg per mL DERA. It seems that it is best to use 7.2 M acetaldehyde 4 stock solution for the feeding at a flow rate of 0.1 μL min^−1^. Under these conditions, about 71 mM phenylacetamide-lactol 5 could be obtained (Fig. 6G). The remaining acetaldehyde 4 in the reactor after 12 h is in the range of 60 mM according to the model (Fig. 6H). Furthermore, about 12 mM of alcohol 1 was also not converted after 12 h of the reaction. Thus, the reaction should be continued for a certain time without feeding so that the remaining alcohol 1 can be converted. The concentration of trimer 8 at the optimum conditions is 39 mM, which is again close to the minimum values (Fig. 6I) together with the concentration of the acid 6 at approx. 12 mM (Fig. 6J).

All described simulations were done at 5 mM of NAD^+^. Further simulations (Fig. 6K) revealed that at a value of 2 mM, the concentration of phenylacetamide-lactol 5 can be increased further up to nearly 75 mM, whereby the concentrations of trimer 8 (34.8 mM) and acid 6 (6.4 mM) are somewhat reduced compared to the previous conditions.

The manipulation of the process conditions, based on the knowledge of the mathematical model and the understanding of the complex interactions within the cascade compounds and involved enzymes, resulted in an improvement compared to the batch reactor. The best result in the fed-batch reactor was 75% yield of phenylacetamide-lactol 5 and a reaction selectivity of 1.82. The reaction selectivity was calculated as the ratio between phenylacetamide-lactol 5 concentration and the sum of the concentrations of acid 6 and trimer 8 as by-products. To assess the process suitability for industrial application, the economic aspect of biocatalytic synthesis must be considered. A good key figure for the early economic evaluation of a process is the biocatalyst yield, which represents the ratio of product to biocatalyst mass.^67^ In the case of a fed-batch reactor using the conditions for the highest phenylacetamide-lactol 5 concentration, *i.e.*, 75 mM, the biocatalyst yield was 0.32 g_of 5_ g_biocatalysts_^−1^. Literature shows^68^ that for the pharma and fine chemicals industry biocatalyst yield should be in the range of 10 to 100 g_product_ g_biocatalyst_^−1^ which implies that the biocatalyst yield in the investigated reaction needs to be significantly improved prior to industrial application. In other words, the studied cascade does not yet represent a system that can be transferred to an industrial scale. However, the findings on the limits of the enzymes and the knowledge of the complex interactions in the cascade system provide valuable information on properties that should be improved. As the influence of inhibitors has been shown to be the main obstacle for obtaining better process metrics, future research should focus on the discovery and design of enzymes with better specificity and activity to avoid byproduct formation and the required enzyme concentration. Moreover, to approach the required thresholds of economic parameters for industrial viability, whole-cell biocatalysis with all enzymes could be considered, which can significantly reduce the cost of catalyst production compared to the purification of multiple enzymes.

The investigated cascade reaction represents an interesting alternative for the synthesis of statin side chain precursor. Further research should be directed towards its advancement and optimization to reach industrial viability. Additionally, a preliminary value-added evaluation was carried out to emphasize the attractiveness of this approach, in contrast to starting with the aldol addition, which was also previously studied at a different pH value.^53^ The presented approach, starting from 3-aminopropanol instead of 3-aminopropanal, allows a significant cost reduction of the starting material (Table 3). Using the lower prices of 3-aminopropanol (60 $ for 1 kg (AbaChemScene)), raw material costs for the synthesis of *N*-(3-oxopropyl)-2-phenylacetamide (aldehyde 2) *via* the chemical synthesis of protected alcohol (starting from 3-aminopropanol to obtain *N*-(3-hydroxypropyl)-2-phenylacetamide (1)) and its enzymatic oxidation in the cascade reaction (oxidation of *N*-(3-hydroxypropyl)-2-phenylacetamide (1) to *N*-(3-oxopropyl)-2-phenylacetamide (2)) per year were calculated (details of the calculations can be found in ESI, S7†). The calculations were performed for 10 tons of product (both *N*-(3-hydroxypropyl)-2-phenylacetamide and *N*-(3-oxopropyl)-2-phenylacetamide) per year. It was assumed that the yield for both steps, chemical synthesis and enzymatic oxidation, would total 75%, which corresponds to the results presented in this study. In the calculations for the oxidation, the cost of the coenzyme (NAD^+^) and both enzymes (ADH and NOX) were considered, while the cost of the buffer was neglected because the cascade reaction takes place in one pot and no extraction of the aldehyde is performed. The calculations have shown that the price of the enzymes, especially ADH, is the largest part of the final cost. The price of ADH accounts for 92% of the total cost. The reason for this is the assumption that commercially available ADH would be used. The price of this enzyme could be reduced if it were produced in-house. Using these calculations, the price of *N*-(3-oxopropyl)-2-phenylacetamide (2) produced in this manner (in a cascade reaction without its isolation) was calculated to be 103.4 $ per g which is lower than commercial prices (*e.g.*, 4013 $ for 10 g, Enamine Ltd). From these preliminary calculations, it can be concluded that this alternative for the synthesis of statin side chain precursor is worth pursing and worth optimizing further by searching for improved catalysts.

**Table 3:** Prices of starting chemicals for the production of statin side chain precursor

Chemical	Price/$ per g	Supplier
3-Aminopropanal	288.4	Enamine Ltd
	189.0	Chemazone
3-Aminopropanol	3.2	Enamine Ltd
	0.06	AbaChemScene
*N*-(3-Oxopropyl)-2-phenylacetamide	401.3	Enamine Ltd
*N*-(3-Hydroxypropyl)-2-phenylacetamide	108	Enamine Ltd

## Conclusions

In this work a novel route for the synthesis of statin side chain precursor was studied by applying a reaction engineering approach. The synthetic route is a complex cascade reaction, which could not be comprehended extensively without a detailed investigation of the reaction kinetics. The reaction kinetics gives valuable insights into the process, *e.g.*, our findings have shown that the investigated reaction should be performed as a cascade with all reactions occurring simultaneously to minimize the negative effect of aldehyde 2 on the oxidation. Additionally, acetaldehyde 4 should be continuously fed into the reactor to keep its concentration at a minimum because it causes inhibition and side reactions such as dimerization 7 and trimerization 8. Findings such as these focus the experimental work and direct it to the correct reactor mode by narrowing down the choices. Furthermore, it is possible to limit the space of the concentrations of substrates and enzymes investigated by performing experiments only *in silico*, thus reducing the time and cost of experiments.

The mathematical model presented in this paper shows the complexity of the system, as there are many dependencies and several side reactions. The findings from the applied mathematical model and the identified enzyme constraints allowed an important improvement of the process metrics. In the best case, when a fed-batch reactor was used under carefully selected process conditions, a 75% yield of phenylacetamide-lactol 5 and a reaction selectivity of 1.82 could be achieved. The reaction engineering approach provided us with valuable insight into the process interdependencies and a deeper understanding of its limitations, which can serve as a solid basis for the investigation and optimization of similar systems.

## List of symbols and abbreviations

*c*
: molar concentration, mM
*k*_d_
: enzyme operational stability decay rate constants, min^−1^
*K*_i_
: inhibition constant, mM
*K*_m_
: Michaelis constant, mM
*r*
: reaction rate, mM min^−1^
S.A.: specific activity, U mg^−1^
*t*
: reaction time, min
*V*_enz_
: enzyme volume, mL
*V*_m_
: maximum reaction rate, U mg^−1^
*V*_r_
: reactor volume, mL
*q*
: volume flow rate, μL min^−1^
*γ*
: mass concentration, mg mL^−1^
acid 6: *N*-phenylacetyl-β-alanine
ADH: alcohol dehydrogenase
alcohol 1: *N*-(3-hydroxypropyl)-2-phenylacetamide
aldehyde 2: *N*-(3-oxopropyl)-2-phenylacetamide
DERA: 2-deoxyribose-5-phosphate aldolase
dimer 7: 3-hydroxybutanal
HPLC: high performance liquid chromatography
NOX: NADH oxidase
phenylacetamide-lactol 5: *N*-(2-((2*S*,4*S*,6*S*)-4,6-dihydroxytetrahydro-2*H*-pyran-2-yl)ethyl)-2-phenylacetamide
TEA HCl: triethanolamine hydrochloride
TFA: trifluoroacetic acid
trimer 8: 3,5-dihydroxyhexanal

## Data availability

The data supporting this article have been included as part of the ESI.†

## Author contributions

Martina Sudar: conceptualization, formal analysis, investigation, methodology, validation, visualization, writing – original draft. Nevena Milčić: investigation, methodology, writing – review & editing. Morana Česnik Katulić: investigation, methodology, writing – review & editing. Anna Szekrenyi: resources. Karel Hernández: methodology, resources. Melinda Fekete: methodology, resources. Rainer Wardenga: resources, writing – review & editing. Maja Majerić Elenkov: methodology, resources, writing – review & editing. Yuyin Qi: methodology, resources. Simon Charnock: resources, writing – review & editing. Đurđa Vasić-Rački: methodology, supervision, writing – review & editing. Wolf-Dieter Fessner: funding acquisition, project administration, resources, writing – review & editing. Pere Clapés: methodology, resources, writing – review & editing. Zvjezdana Findrik Blažević: conceptualization, funding acquisition, methodology, project administration, resources, validation, visualization, supervision, writing – original draft.

## Conflicts of interest

There are no conflicts to declare.

## Supplementary Material

RA-014-D4RA01633E-s001
