# Supplementary material for: Cascade enzymatic synthesis of a statin side chain precursor – the role of reaction engineering in process optimization
Source: RSC Adv. 2024 Jul 4;14(29):21158–73. doi: 10.1039/d4ra01633e (PMC11223575; doi:10.1039/d4ra01633e)
Supplement: RA-014-D4RA01633E-s001 [file RA-014-D4RA01633E-s001.pdf]

Electronic Supplementary Material (ESI) for RSC Advances.
This journal is © The Royal Society of Chemistry 2024

**Supplementary information**

# Cascade enzymatic synthesis of a statin side chain precursor – the role of reaction engineering in process optimization

Martina Sudar,[1,*] Nevena Milčić,[1] Morana Česnik Katulić,[1] Anna Szekrenyi,[2] Karel Hernández,[3] Melinda Fekete,[4, 5] Rainer Wardenga,[4] Maja Majerić Elenkov,[6] Yuyin Qi,[7] Simon Charnock,[7] Đurđa Vasić-Rački,[1] Wolf-Dieter Fessner[2], Pere Clapés,[3] Zvjezdana Findrik Blažević[1,*]

[1] University of Zagreb Faculty of Chemical Engineering and Technology, Savska c. 16, HR-10000 Zagreb, Croatia

[2] Technische Universität Darmstadt, Peter-Grünberg-Straße 4, 64287 Darmstadt, Germany

[3] Institute of Advanced Chemistry of Catalonia, Biotransformation and Bioactive Molecules Group, IQAC-CSIC, Jordi Girona 18-26, 08034 Barcelona, Spain

[4] Enzymicals AG, Walther-Rathenau-Straße 49b, 17489 Greifswald, Germany

[5] piCHEM Forschungs- und Entwicklungs GmbH, Parkring 3, 8074 Raaba-Grambach, Austria

[6] Ruđer Bošković Institute, Bijenička cesta 54, HR-10 000 Zagreb, Croatia

[7] Prozomix Ltd, Haltwhistle, Northumberland, NE49 9HA United Kingdom

[*]Corresponding authors:
e-mail: msudar@fkit.unizg.hr, tel: +385 1 4597 101, fax: +385 1 4597 133
e-mail: zfindrik@fkit.unizg.hr, tel: +385 1 4597 157, fax: +385 1 4597 133

**Contents**

*S1. Synthesis and purification of N-(3-hydroxypropyl)-2-phenylacetamide (**1**)*
*S2. Synthesis and purification of N-(3-oxopropyl)-2-phenylacetamide (**2**)*
*S3. Kinetic investigations of each reaction step of the cascade reaction*
*S4. Operational stability of enzymes in the reactor*
*S5. Statistical analysis of the simulated experiments*
*S6. Fed-batch reactor scheme*
*S7. Raw material costs calculations*

*S8. Synthesis and characterization of N-(2-((2R,4R)-4,6-dihydroxytetrahydro-2H-pyran-2-yl)ethyl)-2-phenylacetamide (**5**)*
*S9. HPLC chromatograms and NMR spectra*

***S1. Synthesis and purification of N-(3-hydroxypropyl)-2-phenylacetamide (1)***

The synthesis of the title compound was performed following the procedure described in the literature (Calveras et al., 2006). The crude product was purified by column chromatography on silica gel using dichloromethane/methanol (97:3) as eluent. Pure alcohol **1** was isolated in 75% yield (4.68 g of a white solid). $^{1}$H NMR spectrum was consistent with the one reported in the literature (Iley et al., 1998).

***S2. Synthesis and purification of N-(3-oxopropyl)-2-phenylacetamide (2)***

The synthesis of the title compound was performed according to the literature (Calveras et al., 2006). The crude product was purified by column chromatography on silica gel using dichloromethane/ethyl acetate (1:1) as eluent. Pure aldehyde **2** was isolated in 21% yield (1.3 g of a white solid). $^{1}$H NMR spectrum was consistent with the one reported in the literature (Calveras et al., 2006). $^{1}$H NMR (400 MHz, D2O) d 9.73 (t, J = 1.5x2 Hz, 1H), 7.32 (m, 5H), 3.49 (s, 2H), 2.66 (t, *J* = 6.3x2 Hz, 2H). $^{13}$C NMR (101 MHz, $D_2O$) d 205.6, 174.5, 134.9, 128.9, 42.5, 42.2, 33.0.

***S3. Kinetic investigations of each reaction step of the cascade reaction***

The kinetics of the reaction was investigated for each reaction step in the cascade. The first step was the oxidoreduction of *N*-(3-hydroxypropyl)-2-phenylacetamide (**1**) to *N*-(3-oxopropyl)-2-phenylacetamide (**2**) catalyzed ADH (Scheme 1). The kinetics of the oxidation of **1** in the presence of $NAD^+$, and the kinetics of the reduction of **2** in the presence of NADH were investigated in detail. These results are presented and explained in chapters S3.1. and S3.2. The oxidoreduction is carried out with coenzyme regeneration. Thus, the kinetics of the $NAD^+$ coenzyme regeneration system catalyzed by NOX is presented in S3.3. As acetaldehyde **4** is one of the substrates, and it was expected that its conversion by ADH to ethanol **3** is enabled, the kinetics of this reversible reaction was also investigated in detail. It was necessary to assess if this reaction can significantly affect the reaction outcome, and if the cascade system could additionally benefit from the coenzyme regeneration induced by this reaction. The results are presented and discussed in chapters S3.4. and S3.5 for acetaldehyde reduction and ethanol oxidation catalyzed by ADH, respectively. The kinetics of aldol addition of **4** to **2** catalyzed by DERA is presented in chapter S3.6. It was found during the cascade experiments that acid **6** is formed. It was assumed that the reaction is enzyme-catalyzed, and the kinetics of this reaction was evaluated and investigated with ADH, DERA, as well as NOX. The results are presented

in chapters S3.7.-S3.9, respectively. As DERA catalyzes the dimerization and trimerization of acetaldehyde, rendering products **7** and **8**, respectively, these reactions were also investigated and results presented in chapters S3.10 and S3.11, respectively.

***S3.1. Kinetics of N-(3-hydroxypropyl)-2-phenylacetamide oxidation catalyzed by ADH***

The influence of the concentrations of substrates, i.e., alcohol **1** and $NAD^+$, on the specific enzyme activity in the oxidation of alcohol **1** is presented in Figs. S1A and B. Both figures show a clear Michaelis-Menten dependence. The reaction is inhibited by both products, i.e., aldehyde **2** and NADH, which is presented in Figs. S1C and D. The influence of the concentrations of acetaldehyde **4** and ethanol **3**, which will be present in the cascade reaction was also evaluated and is presented in Figs. S1E and F, respectively. Acetaldehyde shows an inhibiting effect on the enzyme, but this was not so for ethanol **3**.

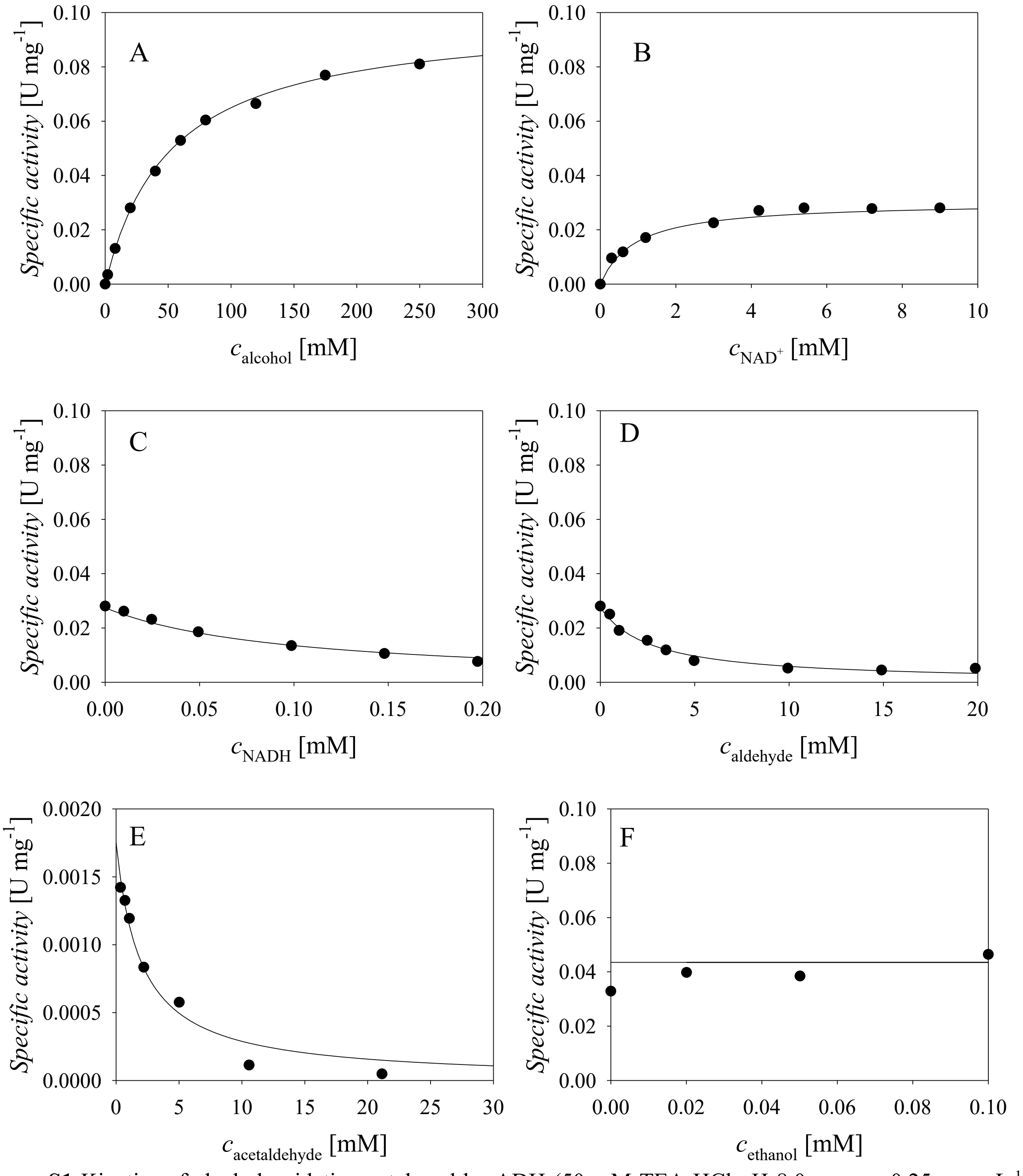


**Figure S1** Kinetics of alcohol oxidation catalyzed by ADH (50 mM TEA HCl pH 8.0, $\gamma_{ADH}$ = 0.25 mg mL$^{-1}$). Dependence of ADH specific activity on the concentration of **A**. alcohol ($c_{NAD^+}$ = 8.99 mM), **B**. NAD$^+$ ($c_{alcohol}$ = 19.98 mM), **C**. NADH ($c_{alcohol}$ = 19.98 mM, $c_{NAD^+}$ = 8.99 mM), **D**. aldehyde ($c_{alcohol}$ = 19.98 mM, $c_{NAD^+}$ = 8.99 mM), **E**. acetaldehyde ($c_{alcohol}$ = 10.35 mM, $c_{NAD^+}$ = 9.04 mM), **F**. ethanol ($c_{alcohol}$ = 19.98 mM, $c_{NAD^+}$ = 8.99 mM).

The experimental data in Fig. S1 were used to estimate the values of the kinetic parameters of the oxidation which is presented in Table S1. Their values will be commented in the next chapter in the context of the equilibrium reaction catalyzed by ADH.

### *S3.2. Kinetics of N-(3-oxopropyl)-2-phenylacetamide reduction catalyzed by ADH*

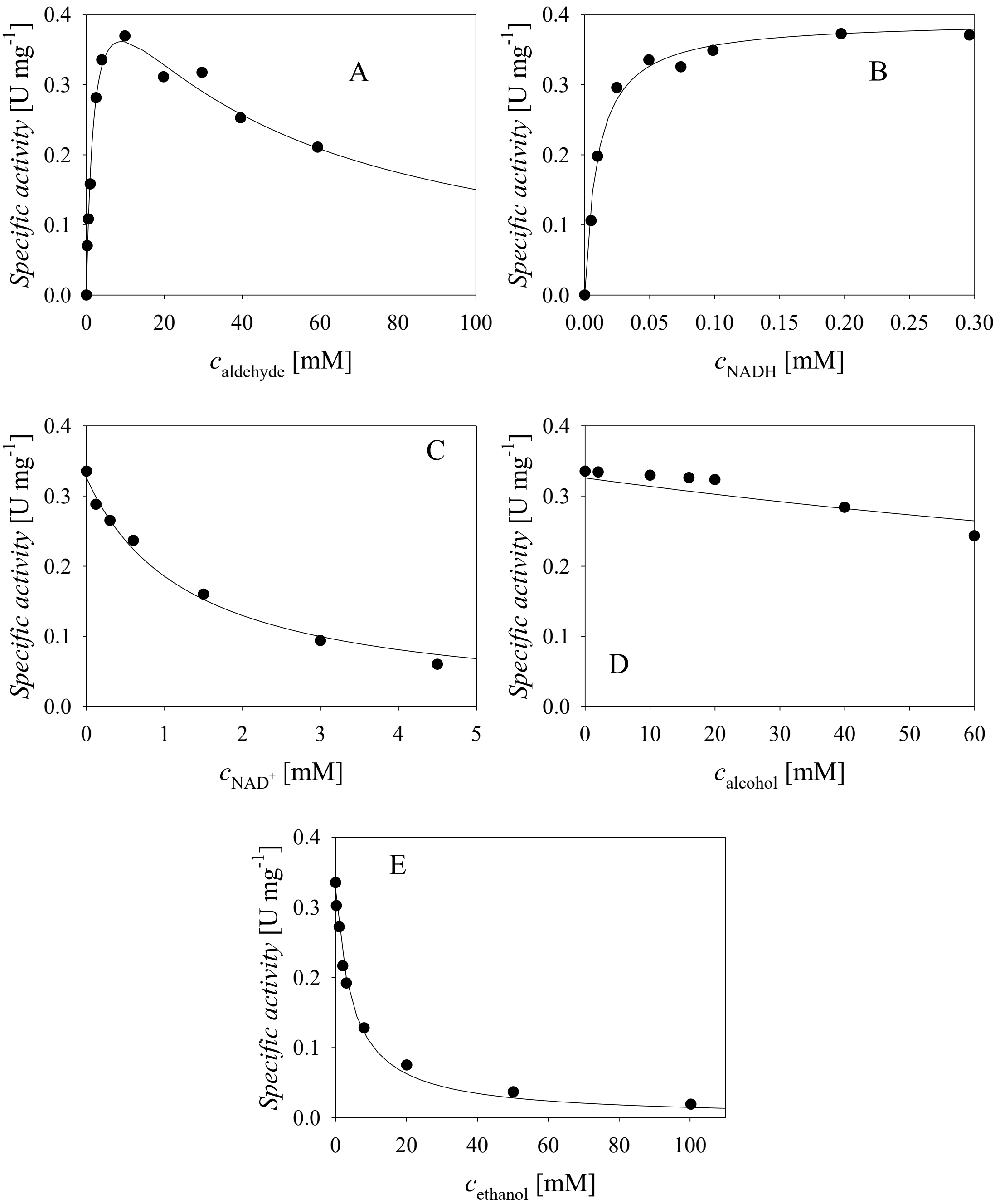


**Figure S2** Kinetics of aldehyde reduction catalyzed by ADH (50 mM TEA HCl pH 8.0, $\gamma_{ADH}$ = 0.05 mg mL$^{-1}$). Dependence of ADH specific activity on the concentration of **A**. aldehyde ($c_{NADH}$ = 0.05 mM), **B**. NADH ($c_{aldehyde}$ = 3.96 mM), **C**. NAD$^+$ ($c_{aldehyde}$ = 3.96 mM, $c_{NADH}$ = 0.05 mM), **D**. alcohol ($c_{aldehyde}$ = 3.95 mM, $c_{NADH}$ = 0.05 mM), **E**. ethanol ($c_{aldehyde}$ = 3.95 mM, $c_{NADH}$ = 0.05 mM).

The influence of the concentrations of substrates in the reduction of aldehyde **2** catalyzed by ADH, i.e., aldehyde **2** and NADH, is presented in Figs. S2A and B, respectively. The enzyme is substrate-inhibited by aldehyde **2**, and for the influence of NADH, Michaelis-Menten dependence was obtained. From the results presented in Fig. S2C and D it can be observed that

both $NAD^+$ and alcohol **1** inhibit the enzyme in this reduction. Fig. S2E shows that ethanol **3** inhibits the reaction as well, while the influence of acetaldehyde **4** concentration could not be measured. It is a competing reaction with the investigated-one, and its rate was higher than the rate of aldehyde **2** reduction, even at concentrations of acetaldehyde as low as 2 mM. So, the inhibition by acetaldehyde **4** could not have been measured like this.

**Table S1** Estimated kinetic parameters of ADH-catalyzed oxidoreduction.

| Parameter | Unit | Value |
|---|---|---|
| **Alcohol oxidation** | | |
| $V_{m1}$ | U mg$^{-1}$ | 0.109 ± 0.001 |
| $K_{m1}^{alcohol}$ | mM | 51.919 ± 2.263 |
| $K_{m1}^{NAD+}$ | mM | 0.916 ± 0.121 |
| $K_{i1}^{NADH}$ | µM | 8.987 ± 0.643 |
| $K_{i1}^{aldehyde}$ | mM | 1.967 ± 0.171 |
| $K_{i1}^{acetaldehyde}$ | mM | 1.646 ± 0.414 |
| **Aldehyde reduction** | | |
| $V_{m2}$ | U mg$^{-1}$ | 0.626 ± 0.007 |
| $K_{m2}^{aldehyde}$ | mM | 1.981 ± 0.341 |
| $K_{i2}^{aldehyde}$ | mM | 40.950 ± 7.592 |
| $K_{m2}^{NADH}$ | µM | 9.969 ± 1.168 |
| $K_{i2}^{NAD+}$ | mM | 0.222 ± 0.015 |
| $K_{i2}^{ethanol}$ | mM | 1.493 ± 0.095 |
| $K_{i2}^{alcohol}$ | mM | 81.240 ± 19.868 |

All estimated kinetic parameters are presented in Table S1. By looking at their values it can be observed that the rate of reduction ($V_{m2}$) is ca six-fold higher than the oxidation ($V_{m1}$), meaning that, as expected, equilibrium shift is necessary. This can be achieved by coenzyme regeneration, but also by in situ aldehyde consumption in the aldol addition. Still, the kinetics is not that simple, considering many inhibitions that occur in this oxidoreduction. In the case of alcohol **1** oxidation all inhibitions are severe, which is substantiated by low values of inhibition constants for NADH ($K_{i1}^{NADH}$), aldehyde **2** ($K_{i1}^{aldehyde}$) and acetaldehyde **4** ($K_{i1}^{acetaldehyde}$). The latter is of biggest concern considering that we want to perform a one pot cascade reaction including both oxidation and aldol addition taking place at the same time. Thus, acetaldehyde **4** needs to be added slowly in a fed-batch mode. As far as aldehyde **2** reduction is concerned there is a mild substrate inhibition ($K_{i2}^{aldehyde}$), and inhibition by alcohol **1** ($K_{i2}^{alcohol}$), and more severe inhibitions by $NAD^+$ ($K_{i2}^{NAD+}$) and ethanol **3** ($K_{i2}^{ethanol}$).

### *S3.3. Kinetics of NAD⁺ regeneration catalyzed by NOX*

The influence of the concentrations of substrates, i.e., NADH and oxygen, on the specific activity of NOX is presented in Figs. S3A and B. They show typical Michaelis-Menten behavior. The reaction is inhibited by $NAD^+$ (Fig. S3C), ethanol **4** (Fig. S3D), acetaldehyde **3** (Fig. S3D), alcohol **4** (Fig. S3E) and aldehyde **2** (Fig. S3F). In efficient coenzyme regeneration $NAD^+$ concentration will be at its maximum in the reactor, which will also bring benefit to the oxidation of alcohol **1** considering that the reverse reaction is severely inhibited by $NAD^+$.

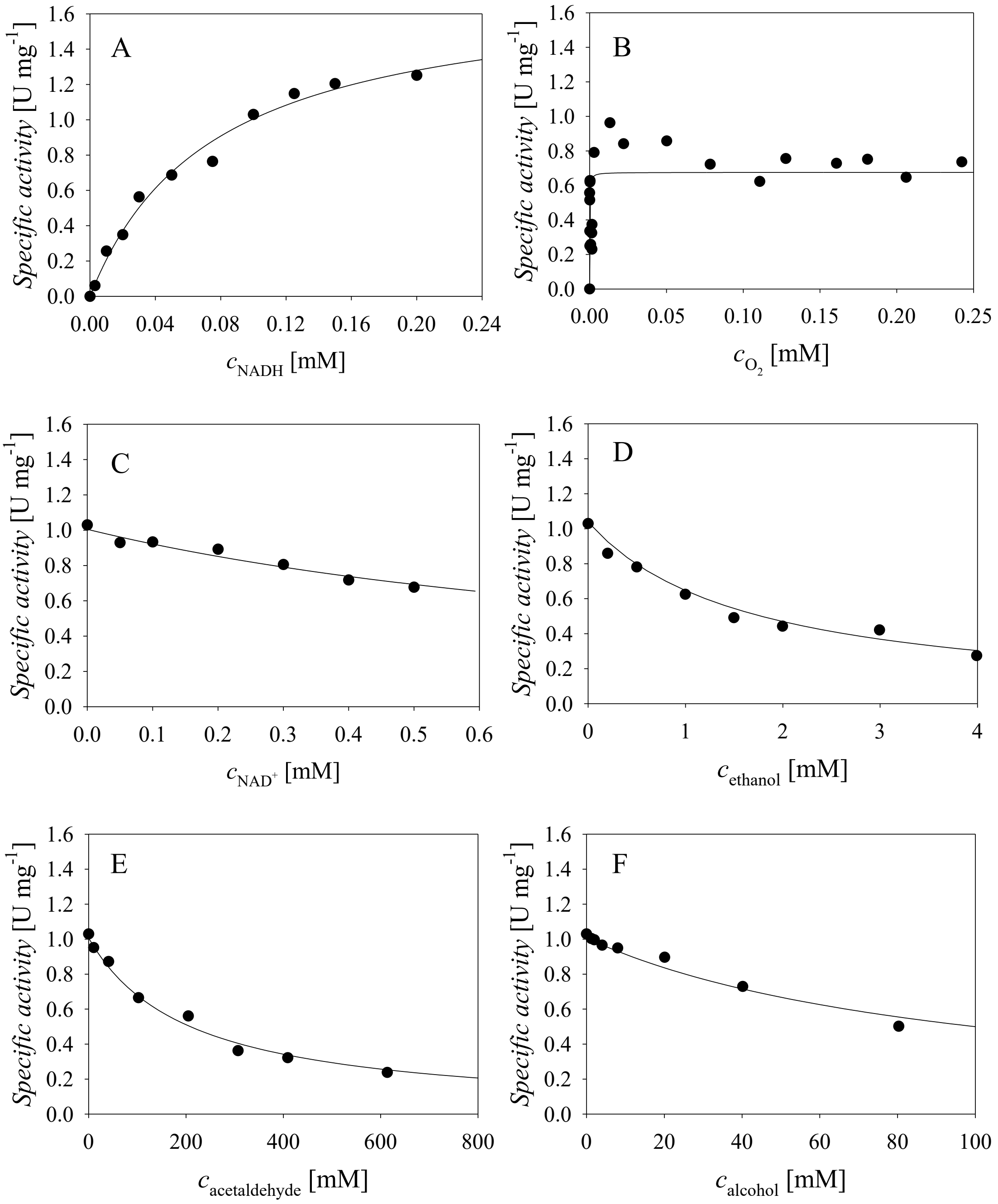

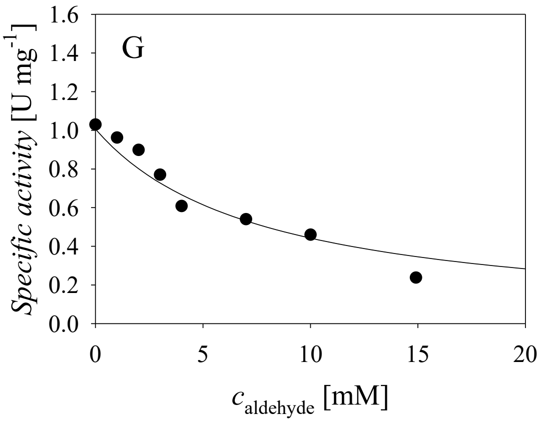


**Figure S3** Kinetics of $NAD^+$ regeneration catalyzed by NOX (50 mM TEA HCl pH 8.0, $\gamma_{NOX}$ = 0.03 mg $mL^{-1}$). Dependence of NOX specific activity on the concentration of **A**. NADH, **B**. $O_2$ ($c_{NADH}$ = 0.1 mM), **C**. $NAD^+$ ($c_{NADH}$ = 0.1 mM), **D**. ethanol ($c_{NADH}$ = 0.1 mM), **E**. acetaldehyde ($c_{NADH}$ = 0.1 mM), **F**. alcohol ($c_{NADH}$ = 0.1 mM), **G**. aldehyde ($c_{NADH}$ = 0.1 mM).

**Table S2** Estimated kinetic parameters for NOX-catalyzed coenzyme regeneration.

| **Parameter** | **Unit** | **Value** |
|---|---|---|
| **Coenzyme regeneration** | | |
| $V_{m3}$ | U $mg^{-1}$ | 1.761 ± 0.115 |
| $K_{m3}^{NADH}$ | mM | 0.075 ± 0.012 |
| $K_{m3}^{O_2}$ | μM | 0.091 ± 0.072 |
| $K_{i3}^{NAD^+}$ | mM | 0.630 ± 0.045 |
| $K_{i3}^{ethanol}$ | mM | 0.678 ± 0.079 |
| $K_{i3}^{acetaldehyde}$ | mM | 77.326 ± 5.315 |
| $K_{i3}^{aldehyde}$ | mM | 3.361 ± 0.414 |
| $K_{i3}^{alcohol}$ | mM | 42.265 ± 3.874 |

The estimated kinetic parameters for $NAD^+$ regeneration are presented in Table S2. The Michaelis constant for oxygen is extremely low ($K_{m3}^{O_2}$) which indicates that NOX requires minimum amount of oxygen for achieving maximum reaction rate. In practice, this means that the oxygen concentration in the reactor can be low, and aeration may not be required for the process to normally run. As far as inhibitions are concerned, it can be concluded that inhibitions by $NAD^+$ ($K_{i3}^{NAD^+}$), ethanol **3** ($K_{i3}^{ethanol}$) and aldehyde **2** ($K_{i3}^{aldehyde}$) are more significant, whereas inhibitions by acetaldehyde ($K_{i3}^{acetaldehyde}$) and alcohol **3** ($K_{i3}^{alcohol}$) are less significant.

### *S3.4. Kinetics of acetaldehyde reduction catalyzed by ADH*

The influence of substrate concentrations, i.e., acetaldehyde **4** and NADH, on the specific activity of ADH in the acetaldehyde **4** reduction is presented in Figs. S4A and B. Acetaldehyde shows mild substrate-inhibiting effect on ADH (Fig. S4A). Reaction products, i.e., ethanol **3** (Fig. S4C) and $NAD^+$ (Fig. S4D), both inhibit the enzyme in this reduction. While alcohol **1**

(Fig. S4E) also inhibits the enzyme in the reduction of acetaldehyde **4**, no inhibition was found for aldehyde **2** (Fig. S4F). The estimated kinetic parameters are presented in Table S3.

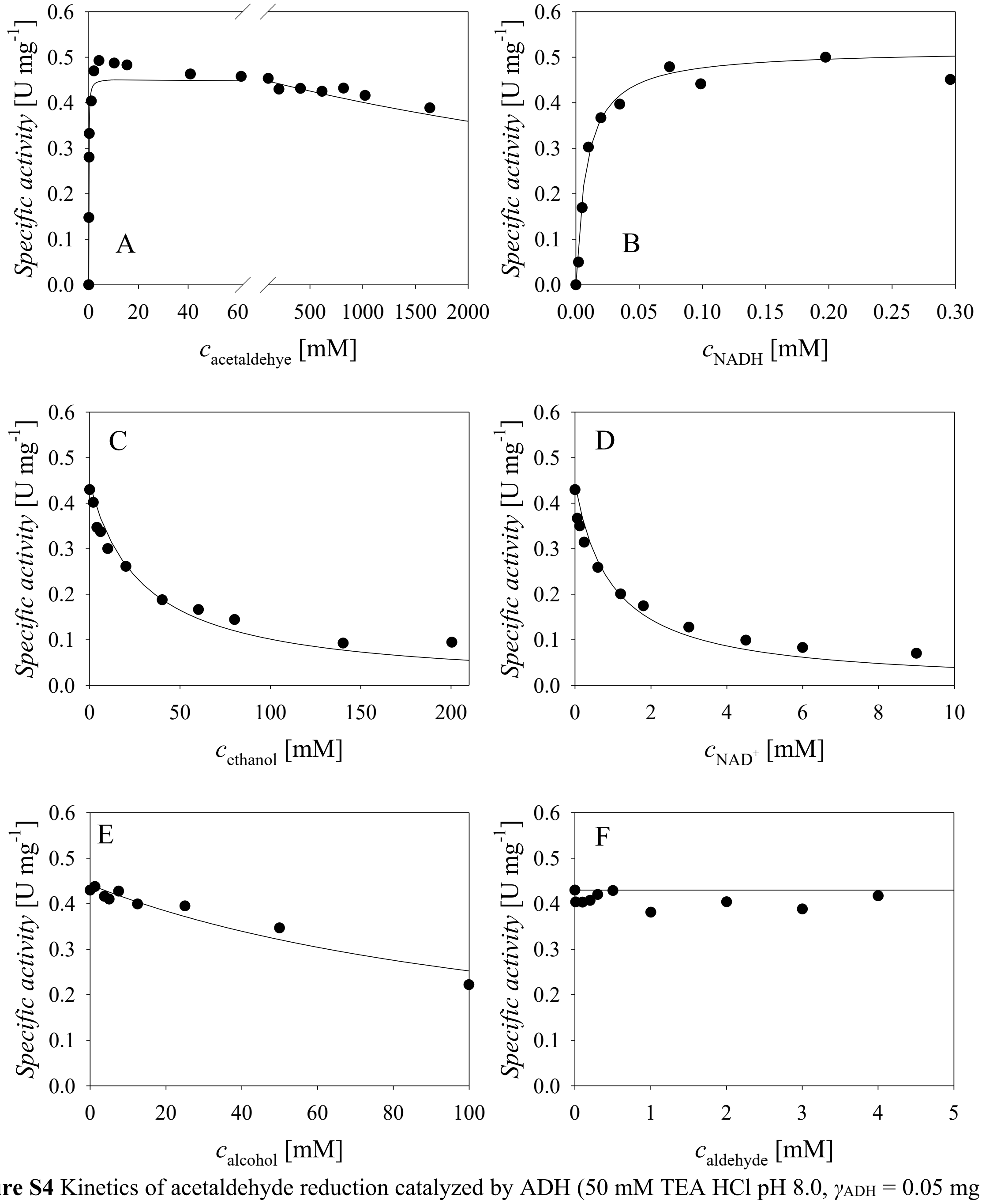


**Figure S4** Kinetics of acetaldehyde reduction catalyzed by ADH (50 mM TEA HCl pH 8.0, $\gamma_{ADH}$ = 0.05 mg mL$^{-1}$). Dependence of ADH specific activity on the concentration of **A**. acetaldehyde ($c_{NADH}$ = 0.05 mM), **B**. NADH ($c_{acetaldehyde}$ = 204.52 mM), **C**. ethanol ($c_{NADH}$ = 0.05 mM, $c_{acetaldehyde}$ = 204.52 mM), **D**. $NAD^+$ ($c_{NADH}$ = 0.05 mM, $c_{acetaldehyde}$ = 204.52 mM), **E**. alcohol ($c_{NADH}$ = 0.05 mM, $c_{acetaldehyde}$ = 204.52 mM), **F**. aldehyde ($c_{NADH}$ = 0.05 mM, $c_{acetaldehyde}$ = 204.52 mM).

### *S3.5. Kinetics of ethanol oxidation catalyzed by ADH*

The influence of ethanol **3** concentration on the specific activity of ADH in the oxidation of ethanol **3** (Fig. S5A) shows its inhibiting effect on enzyme activity. The dependence of $NAD^+$ (Fig. S5B) concentration on the specific activity of ADH can be described by Michaelis-Menten kinetics. Both products of ethanol **3** oxidation, i.e., acetaldehyde **4** (Fig. S5C) and NADH (Fig. S5D), inhibit the enzyme in the oxidation of ethanol **3**. Alcohol **2** (Fig. S5E) shows no effect on enzyme activity in this oxidation while aldehyde **2** inhibits the enzyme (Fig. S5F).

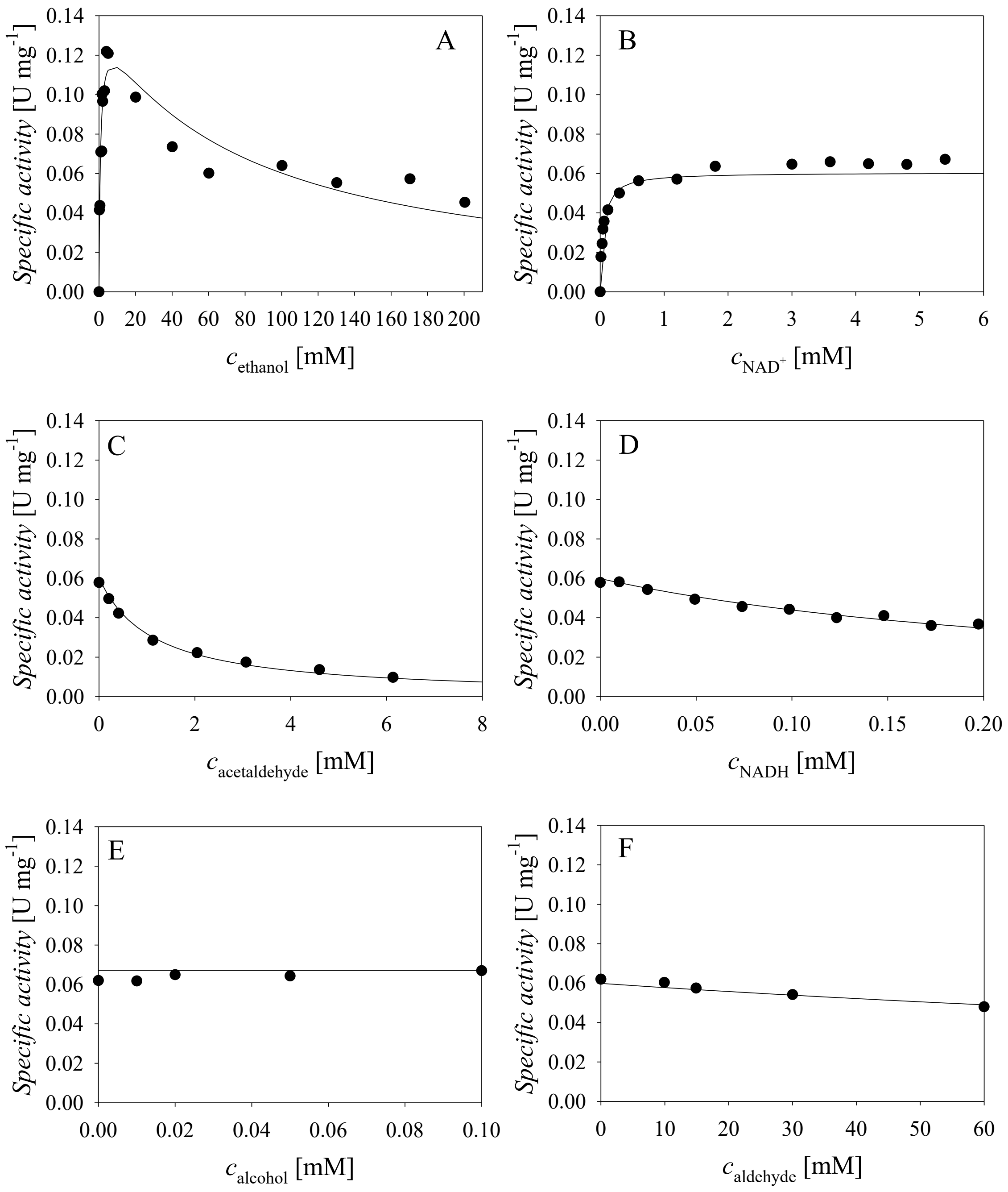

**Figure S5** Kinetics of ethanol oxidation catalyzed by ADH (50 mM TEA HCl pH 8.0, $\gamma_{ADH}$ = 0.125 mg mL$^{-1}$). Dependence of ADH specific activity on the concentration of **A**. ethanol ($c_{NAD^+}$ = 8.99 mM), **B**. NAD$^+$ ($c_{ethanol}$ = 100.22 mM), **C**. acetaldehyde ($c_{ethanol}$ = 100.22 mM, $c_{NAD^+}$ = 4.50 mM), **D**. NADH ($c_{ethanol}$ = 100.22 mM, $c_{NAD^+}$ = 4.50 mM), **E**. alcohol ($c_{ethanol}$ = 100.22 mM, $c_{NAD^+}$ = 4.50 mM), **F**. aldehyde ($c_{ethanol}$ = 100.22 mM, $c_{NAD^+}$ = 4.50 mM).

The estimated kinetic parameters for the acetaldehyde **4** reduction and ethanol **3** oxidation are presented in Table S3. They show that acetaldehyde **4** reduction is favored ($V_{m4}$) in comparison to oxidation ($V_{m5}$), which could also mean that this reduction could serve as an additional NAD$^+$ regeneration system when both oxidation and aldol addition are carried out simultaneously in one pot. ADH shows high affinity towards acetaldehyde **4** (low $K_{m4}^{acetaldehyde}$), mild substrate inhibition (high $K_{i4}^{acetaldehyde}$), and severe inhibitions by NAD$^+$ ($K_{i4}^{NAD+}$) and alcohol **1** ($K_{i4}^{alcohol}$) in the acetaldehyde **4** reduction. At the same time, it shows good affinity towards ethanol **3** ($K_{m5}^{ethanol}$), mild substrate inhibition ($K_{i5}^{ethanol}$), and significant product inhibitions by acetaldehyde **4** ($K_{i5}^{acetaldehyde}$), NADH ($K_{i5}^{NADH}$) and aldehyde **2** ($K_{i5}^{aldehyde}$) in the oxidation of ethanol **3**. All this shows that the reactions catalyzed by ADH make a complex system which needs to be evaluated by in silico experiments to make further assumptions on the reaction outcome.

**Table S3** Estimated kinetic parameters

| **Parameter** | **Unit** | **Value** |
|---|---|---|
| **Acetaldehyde reduction** | | |
| $V_{m4}$ | U mg$^{-1}$ | 0.530 ± 0.010 |
| $K_{m4}^{acetaldehyde}$ | mM | 0.065 ± 0.009 |
| $K_{i4}^{acetaldehyde}$ | mM | 7605.023 ± 1908.241 |
| $K_{m4}^{NADH}$ | µM | 8.333 ± 1.383 |
| $K_{i4}^{ethanol}$ | µM | 9.163 ± 0.955 |
| $K_{i4}^{NAD+}$ | mM | 0.141 ± 0.018 |
| $K_{i4}^{alcohol}$ | mM | 0.041 ± 0.005 |
| **Ethanol oxidation** | | |
| $V_{m5}$ | U mg$^{-1}$ | 0.138 ± 0.003 |
| $K_{m5}^{ethanol}$ | mM | 0.777 ± 0.239 |
| $K_{i5}^{ethanol}$ | mM | 78.892 ± 19.512 |
| $K_{m5}^{NAD+}$ | mM | 0.048 ± 0.006 |
| $K_{i5}^{acetaldehyde}$ | µM | 3.858 ± 0.194 |
| $K_{i5}^{NADH}$ | µM | 2.911 ± 0.129 |
| $K_{i5}^{aldehyde}$ | mM | 0.924 ± 0.153 |

### *S3.6. Kinetics of the aldol addition of acetaldehyde to N-(3-oxopropyl)-2-phenylacetamide catalyzed by DERA*

In the aldol addition of acetaldehyde **4** to aldehyde **2**, two equivalents of acetaldehyde **4** react with aldehyde **1** furnishing *N*-(2-((2*R*,4*R*)-4,6-dihydroxytetrahydro-2*H*-pyran-2-yl)ethyl)-2-2-phenylacetamide (phenylacetamide-lactol **5**). The influence of aldehyde **2** on the reaction rate can be described by the Michaelis-Menten kinetics (Fig. S6A), and the influence of acetaldehyde **4** by Michaelis-Menten kinetics with substrate inhibition (Fig. S6B). Figs. S6C-F show that alcohol **1**, ethanol **3**, $NAD^+$ and trimer **8** inhibit the reaction, respectively. The influence of NADH concentration on this reaction was not investigated while it was assumed that due to efficient coenzyme regeneration, its concentration will be close to zero in this system.

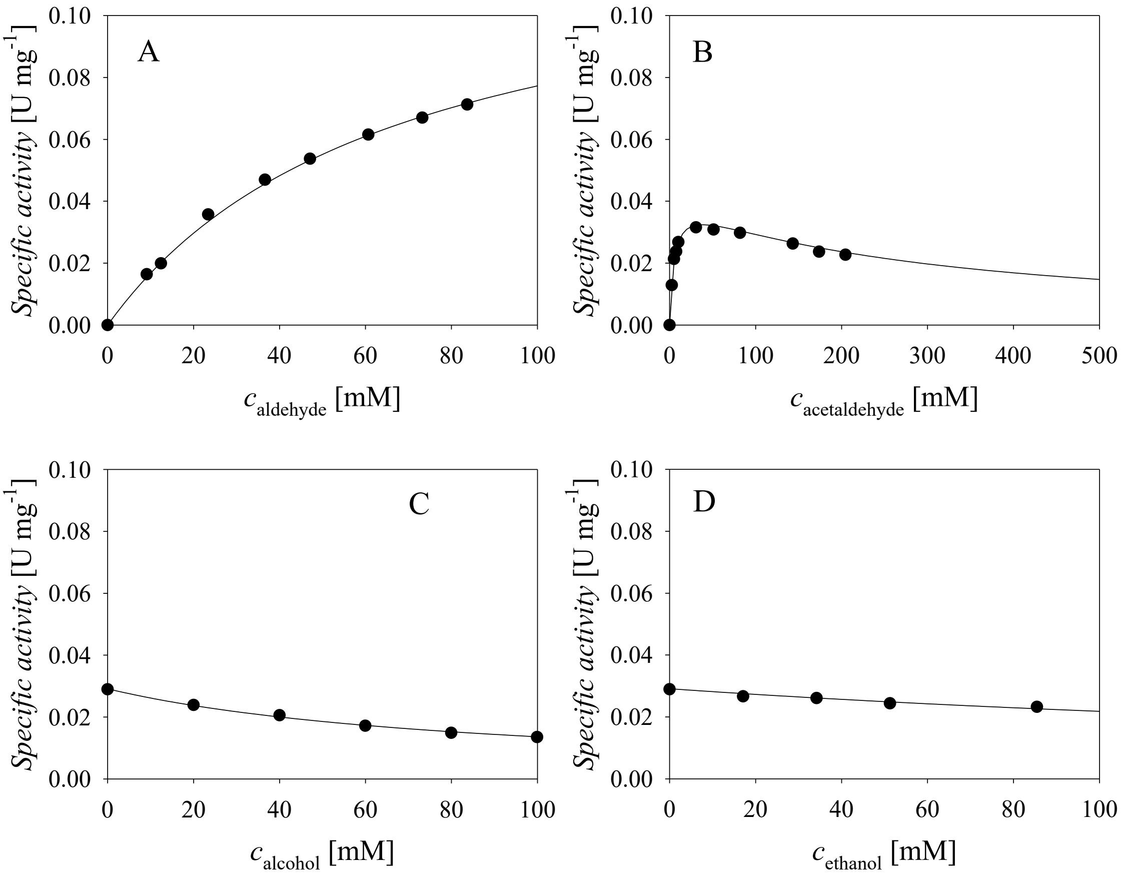

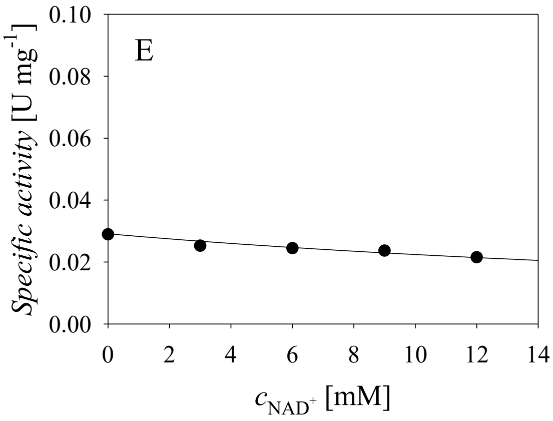


**Figure S6** Kinetics of aldol addition of aldehyde and acetaldehyde catalyzed by DERA (50 mM TEA HCl pH 8.0, $\gamma_{DERA}$ = 10 mg mL$^{-1}$). Dependence of DERA specific activity on the concentration of **A**. aldehyde ($c_{acetaldehyde}$ = 102.26 mM), **B**. acetaldehyde ($c_{aldehyde}$ = 19.50 mM), **C**. alcohol ($c_{aldehyde}$ = 19.50 mM, $c_{acetaldehyde}$ = 102.26 mM), **D**. ethanol ($c_{aldehyde}$ = 19.50 mM, $c_{acetaldehyde}$ = 102.26 mM), **E**. NAD$^+$ ($c_{aldehyde}$ = 19.50 mM, $c_{acetaldehyde}$ = 102.26 mM).

**Table S4** Estimated kinetic parameters for the aldol addition of acetaldehyde to aldehyde

| **Parameter** | **Unit** | **Value** |
|---|---|---|
| **Aldol addition** | | |
| $V_{m6}$ | U mg$^{-1}$ | 0.174 ± 0.007 |
| $K_{m6}^{aldehyde}$ | mM | 67.045 ± 3.143 |
| $K_{m6}^{acetaldehyde}$ | mM | 1.927 ± 0.159 |
| $K_{i6}^{acetaldehyde}$ | mM | 723.802 ± 81.731 |
| $K_{i6}^{alcohol}$ | mM | 68.103 ± 1.556 |
| $K_{i6}^{ethanol}$ | mM | 4.872 ± 0.409 |
| $K_{i6}^{NAD+}$ | mM | 26.058 ± 2.486 |
| $K_{i6}^{trimer}$ | mM | 0.641 ± 0.773 |

The estimated kinetic parameters presented in Table S4 show relatively low specific enzyme activity in the aldol addition ($V_{m6}$), relatively high apparent Michaelis constant for aldehyde **2** meaning that higher concentration of this intermediate is required to achieve maximum activity of DERA. Among other inhibitions, the inhibition by trimer **8** is significant ($K_{i6}^{trimer}$), and difficult to avoid.

## *S3.7. Kinetics of N-(3-oxopropyl)-2-phenylacetamide oxidation catalyzed by ADH*

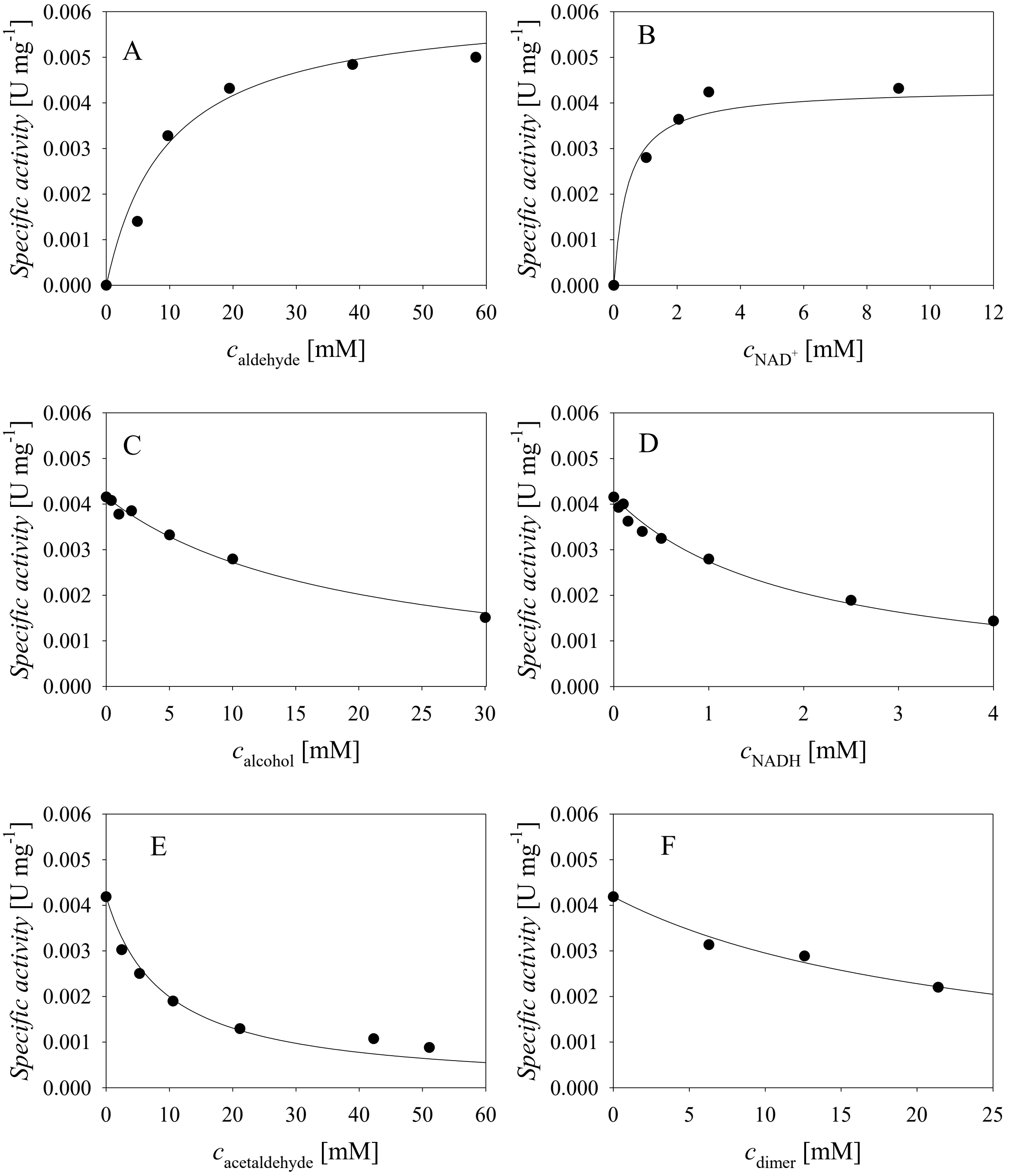


**Figure S7** Kinetics of aldehyde oxidation catalyzed by ADH (50 mM TEA HCl pH 8.0, $\gamma_{ADH}$ = 2.5 mg mL$^{-1}$). Dependence of ADH specific activity on the concentration of **A**. aldehyde ($c_{NAD^+}$ = 9.00 mM), **B**. NAD$^+$ ($c_{aldehyde}$ = 19.45 mM), **C**. alcohol ($c_{aldehyde}$ = 19.87 mM, $c_{NAD^+}$ = 9.00 mM), **D**. NADH ($c_{aldehyde}$ = 19.87 mM, $c_{NAD^+}$ = 9.00 mM), **E**. acetaldehyde ($c_{aldehyde}$ = 19.87 mM, $c_{NAD^+}$ = 10.85 mM), **F**. dimer ($c_{aldehyde}$ = 19.87 mM, $c_{NAD^+}$ = 10.85 mM).

It was observed that during the cascade reaction *N*-phenylacetyl-β-alanine (acid **6**) is formed from aldehyde **2**. Thus, it was necessary to establish the enzyme(s) responsible for that purpose. The kinetics of aldehyde **2** oxidation to acid **6** catalyzed by ADH was investigated, and the results are presented in Fig. S7. The enzyme is active in this biotransformation and shows the

same kinetics as in the case of primary oxidation. Both substrates in this reaction, i.e., aldehyde **2** (Fig. S7A) and $NAD^+$ (Fig. S7B), influence the enzyme activity according to Michaelis-Menten kinetics. The enzyme is inhibited by product NADH (Fig. S7D), and other components of the reaction system, such as alcohol **3** (Fig. S7C), acetaldehyde **4** (Fig. S7E) and dimer **7** (Fig. S7F). The estimated kinetic parameters for this reaction are presented in Table S5, and they show that the enzyme activity in this reaction is quite low ($V_{m7}$), and the apparent affinity of enzyme toward the substrate is moderate ($K_{m7}^{aldehyde}$). There are also few inhibitions, and the most severe is the one by NADH ($K_{i7}^{NADH}$), but others are also significant ($K_{i7}^{alcohol}$, $K_{i7}^{acetaldehyde}$, $K_{i7}^{dimer}$).

**Table S5** Estimated kinetic parameters in the oxidation of aldehyde catalyzed by ADH.

| **Parameter** | **Unit** | **Value** |
|---|---|---|
| **Aldehyde oxidation catalyzed by ADH** | | |
| $V_{m7}$ | U mg$^{-1}$ | 0.006 ± 0.0005 (0.016 ± 0.004) |
| $K_{m7}^{aldehyde}$ | mM | 9.575 ± 2.395 |
| $K_{m7}^{NAD+}$ | mM | 0.434 ± 0.182 |
| $K_{i7}^{alcohol}$ | mM | 6.167 ± 0.371 |
| $K_{i7}^{NADH}$ | mM | 0.089 ± 0.006 |
| $K_{i7}^{acetaldehyde}$ | mM | 2.952 ± 0.313 |
| $K_{i7}^{dimer}$ | mM | 7.783 ± 0.661 |

### *S3.8. Kinetics of N-(3-oxopropyl)-2-phenylacetamide oxidation catalyzed by DERA*

As DERA was supplied as cell-free extract, it was expected that it may also contain dehydrogenase activity responsible for the oxidation of aldehyde **2** to acid **6**. That is why the kinetics of this reaction was also investigated. The results presented in Fig. S8 show that the assumption was right, while the influence of both substrates, i.e., aldehyde **2** and $NAD^+$, on the specific enzyme activity can be described by the Michaelis-Menten kinetics. The estimated kinetic parameters are presented in Table S6, and they show that the maximum reaction rate of this reaction ($V_{m8}$) is five-fold lower than in the case of ADH. That means that the impact of DERA on acid formation is much lower than that of ADH.

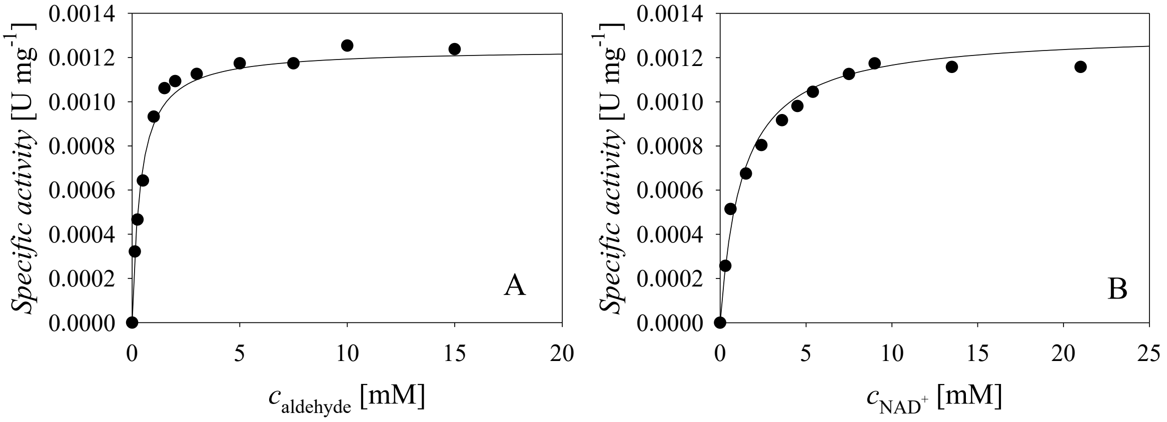


**Figure S8** Kinetics of aldehyde oxidation catalyzed by DERA (50 mM TEA HCl pH 8.0, $\gamma_{DERA}$ = 1 mg mL$^{-1}$). Dependence of DERA specific activity on the concentration of **A**. aldehyde ($c_{NAD^+}$ = 8.99 mM), **B**. NAD$^+$ ($c_{aldehyde}$ = 4.99 mM).

**Table S6** Estimated kinetic parameters in the oxidation of aldehyde catalyzed by DERA.

| **Parameter** | **Unit** | **Value** |
|---|---|---|
| **Aldehyde oxidation catalyzed by DERA** | | |
| $V_{m8}$ | U mg$^{-1}$ | $1.416 \cdot 10^{-3} \pm 3.252 \cdot 10^{-5}$ |
| $K_{m8}^{aldehyde}$ | mM | $0.379 \pm 0.038$ |
| $K_{m8}^{NAD+}$ | mM | $1.289 \pm 0.137$ |

### *S3.9. Kinetics of N-(3-oxopropyl)-2-phenylacetamide oxidation catalyzed by NOX*

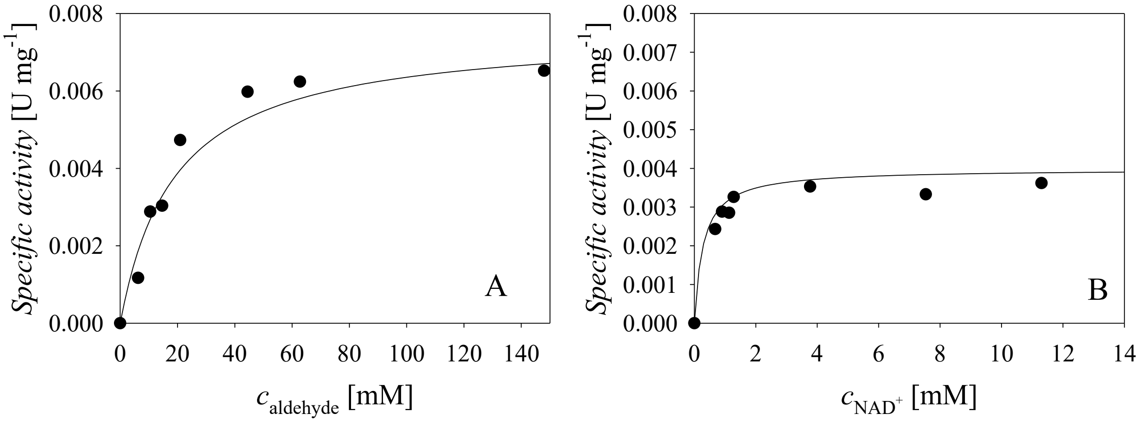


**Figure S9** Kinetics of aldehyde oxidation catalyzed by NOX (50 mM TEA HCl pH 8.0, $\gamma_{NOX}$ = 5 mg mL$^{-1}$). Dependence of NOX specific activity on the concentration of **A**. aldehyde ($c_{NAD^+}$ = 9.04 mM), **B**. NAD$^+$ ($c_{aldehyde}$ = 19.87 mM).

Considering that NOX was also supplied as cell-free extract we evaluated the potential of this enzyme to perform the oxidation of aldehyde **2** to the corresponding acid. The results presented in Fig. S9 show that the enzyme exhibits activity in this reaction. The estimated kinetic parameters presented in Table S7 show that order of magnitude of the maximum reaction rate of the reaction catalyzed by NOX ($V_{m9}$) is very similar to that of ADH ($V_{m7}$). It is thus expected that these two enzymes will have a more significant role in forming the acid, than DERA.

**Table S7** Estimated kinetic parameters of aldehyde oxidation catalyzed by NOX.

| Parameter | Unit | Value |
|---|---|---|
| **Aldehyde oxidation catalyzed by NOX** | | |
| $V_{m9}$ | U mg$^{-1}$ | 0.0078 ± 0.0002 |
| $K_{m9}^{aldehyde}$ | mM | 19.045 ± 4.438 |
| $K_{m9}^{NAD+}$ | mM | 0.282 ± 0.059 |

## *S3.10. Kinetics of acetaldehyde dimerization catalyzed by DERA*

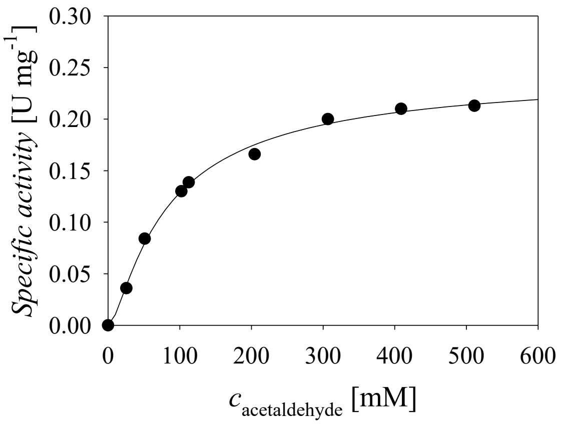


**Figure S10** Kinetics of the side reaction of acetaldehyde dimerization catalyzed by DERA (50 mM TEA HCl pH 8.0, $\gamma_{DERA}$ = 0.1 mg mL$^{-1}$). Dependence of DERA specific activity on the concentration of acetaldehyde.

The influence of acetaldehyde **4** concentration on the specific activity of DERA in the dimerization reaction to furnish **7** was investigated and it was found that it can be described by the Michaelis-Menten kinetics (Fig. S10). In this case, the rate of dimer **7** formation was investigated. The kinetic parameters presented in Table S8 show that the maximum rate of dimerization ($V_{m10}$) is higher than the rate of acetaldehyde **4** addition to aldehyde **2** ($V_{m6}$). This could be influenced by the concentration of acetaldehyde **4** in the reactor, while at lower concentration (Fig. S6B) the rate of aldol addition can be increased, and at the same time the rate of dimerization can be lowered. The rate of dimerization is slightly inhibited by alcohol **1** ($K_{i10}^{alcohol}$) which shows that if higher substrate concentrations are used in the oxidation, the dimerization rate will be lowered.

**Table S8** Estimated kinetic parameters for the acetaldehyde dimerization catalyzed by DERA.

| Parameter | Unit | Value |
|---|---|---|
| **Dimerization of acetaldehyde** | | |
| $V_{m10}$ | U mg$^{-1}$ | 0.248 ± 0.005 |
| $K_{m10}^{acetaldehyde}$ | mM | 39.009 ± 2.074 |
| $K_{i10}^{alcohol}$ | mM | 15.980 ± 5.933 |

## *S3.11. Kinetics of acetaldehyde trimerization catalyzed by DERA*

The reaction between dimer **7** and acetaldehyde **4** in which trimer **8** is formed was further studied. The influence of acetaldehyde **4** and dimer **7** concentrations on the specific enzyme activity can be described by Michaelis-Menten kinetics (Fig. S11). The estimated kinetic parameters are presented in Table S9. The apparent affinity of enzyme towards acetaldehyde **4** in dimerization ($K_{m10}^{acetaldehyde}$) and trimerization ($K_{m11}^{acetaldehyde}$) is significantly lower than in

the case of aldol addition ($K_{m6}^{acetaldehyde}$) which goes in favor of aldol addition. However, to be sure, in silico experiments need to be carried out.

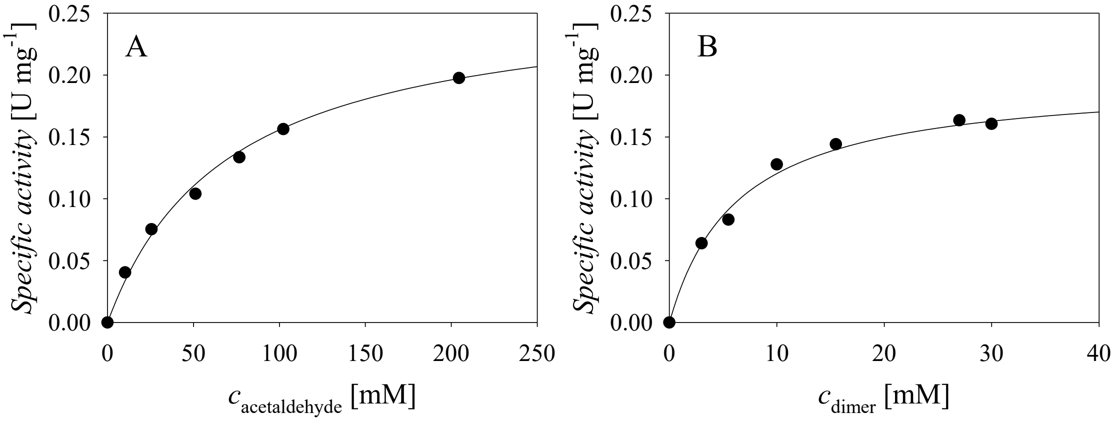


**Figure S11** Kinetics of acetaldehyde trimerization catalyzed by DERA (50 mM TEA HCl pH 8.0, $\gamma_{DERA}$ = 10 mg mL$^{-1}$). Dependence of DERA specific activity on the concentration of **A**. acetaldehyde ($c_{dimer}$ = 25.02 mM), **B**. dimer ($c_{acetaldehyde}$ = 102.26 mM).

**Table S9** Estimated kinetic parameters in acetaldehyde trimerization catalyzed by DERA.

| **Parameter** | **Unit** | **Value** |
|---|---|---|
| **Trimerization of acetaldehyde** | | |
| $V_{m11}$ | U mg$^{-1}$ | 0.332 ± 0.019 |
| $K_{m11}^{acetaldehyde}$ | mM | 69.960 ± 8.070 |
| $K_{m11}^{dimer}$ | mM | 6.388 ± 0.728 |

## *S4. Operational stability of enzymes in the reactor*

**Table S10** Operational stability decay rate constants for enzymes used in the cascade.

| **Enzyme** | $k_d$ / d$^{-1}$ | $t_{1/2}$ / d | |
|---|---|---|---|
| ADH | 1.495 ± 0.201 | 0.464 | exp. in Fig. 1A |
| NOX | 5.246 ± 0.814 | 0.132 | exp. in Fig. 2A |
| DERA | 4.169 ± 0.568 | 0.166 | exp. in Fig. 4 |

## *S5. Statistical output of the SCIENTIST software for the goodness-of-fit of the model to the experimental data.*

The agreement of the experimental data and mathematical model simulations was substantiated by the statistical output of the model calculated in SCIENTIST and is presented in Table S11.

**Table S11** Statistical output of the SCIENTIST software for the goodness-of-fit of the model to the experimental data.

| Figure | $R^2$ | Coefficient of Determination | Correlation | Model Selection Criterion | Standard deviation |
|---|---|---|---|---|---|
| Aldehyde oxidation catalyzed by ADH | | | | | |
| 1A | 0.9980 | 0.9972 | 0.9989 | 5.8186 | 0.2982 |
| 1B | 0.9980 | 0.9961 | 0.9982 | 5.4485 | 0.4854 |
| 1C | 0.9955 | 0.9930 | 0.9968 | 4.8469 | 1.0799 |
| Alcohol oxidation catalyzed by ADH with coenzyme regeneration catalyzed by NOX | | | | | |
| 2A | 0.9715 | 0.9354 | 0.9683 | 2.5857 | 0.9876 |
| 2B | 0.9962 | 0.9933 | 0.9968 | 4.8445 | 1.7069 |
| 2C | 0.9986 | 0.9976 | 0.9993 | 5.8673 | 2.4117 |
| Side reaction of acetaldehyde dimerization and trimerization catalyzed by DERA | | | | | |
| 3A | 0.9765 | 0.9650 | 0.9867 | 3.2327 | 8.1600 |
| 3B | 0.9890 | 0.9833 | 0.9928 | 3.9697 | 9.9415 |
| 3C | 0.9869 | 0.9796 | 0.9906 | 3.8143 | 7.6658 |
| Aldol addition of acetaldehyde to aldehyde catalyzed by DERA | | | | | |
| 4 | 0.9383 | 0.9027 | 0.9555 | 2.3294 | 19.4517 |
| Cascade reaction – cascade model with newly estimated $V_{m7}$ | | | | | |
| 5A | 0.8347 | 0.7474 | 0.8980 | 1.2687 | 14.2413 |
| 5B | 0.8753 | 0.8283 | 0.9271 | 1.6760 | 13.2377 |
| 5C | 0.7050 | 0.5989 | 0.8289 | 0.8265 | 18.0698 |

R-squared is defined by Eq. 1 where $n$ represents the quantity of data points and $w_i$ symbolizes the assigned weights to each individual point. The expression is similar to the equation for the coefficient of determination, where the sum of the squared observed values occupies a comparable position to the role of variance in the equation for the coefficient of determination.

$$R^2 = \frac{\left| \sum_{i=1}^{n} w_i \cdot {Y_{obs_i}}^2 - \sum_{i=1}^{n} w_i \left( Y_{obs_i} - Y_{cal_i} \right)^2 \right|}{\sum_{i=1}^{n} w_i \cdot {Y_{obs_i}}^2} \quad (1)$$

The coefficient of determination is defined by Eq. 2. In this equation $n$ signifies the number of points, $w_i$ stands for the assigned weights to each point and $\overline{Y}_{obs}$ is the weighted average of the observed data. The coefficient of determination is a measure of the fraction of the total variance accounted for by the model and is an appropriate measure of the goodness-of-fit.

$$\text{Coefficient of determination} = \frac{\sum_{i=1}^{n} w_i \left( Y_{obs_i} - \overline{Y}_{obs} \right)^2 - \sum_{i=1}^{n} w_i \left( Y_{obs_i} - Y_{cal_i} \right)^2}{\sum_{i=1}^{n} w_i \left( Y_{obs_i} - \overline{Y}_{obs} \right)^2} \quad (2)$$

The correlation between two variables $X$ and $Y$ is defined by Eq. 3 where $\bar{x}$ and $\bar{y}$ denote the weighted means of $X$ and $Y$, $n$ is the number of points, and $w_i$ signifies the assigned weights to the data points. This correlation may indicate how the changes in one variable are correlated with changes in the other. The correlation value provided by SCIENTIST represents the correlation between the observed and computed values of the dependent variables.

$$\text{Correlation} = \frac{\sum_{i=1}^{n} w_i \left(x_i - \bar{x}\right)\left(y_i - \bar{y}\right)}{\sqrt{\sum_{i=1}^{n} w_i \left(x_i - \bar{x}\right)^2} \sqrt{\sum_{i=1}^{n} w_i \left(y_i - \bar{y}\right)^2}} \tag{3}$$

Model Selection Criterion (MSC) is defined by Eq. 4.

$$MSC = \ln\left(\frac{\sum_{i=1}^{n} w_i \left(Y_{obs_i} - \bar{Y}_{obs}\right)^2}{\sum_{i=1}^{n} w_i \left(Y_{obs_i} - \bar{Y}_{cal_i}\right)^2}\right) - \frac{2p}{n} \tag{4}$$

This criterion is based upon a modified version of the Akaike Information Criterion (AIC). The standard AIC is contingent on the magnitude of data points and the quantity of observations. The model deemed most suitable by AIC is the one with the lowest AIC value. In the case of SCIENTIST, a revised AIC termed MSC is employed. The model offering the best fit is the one associated with the highest MSC value.

The standard deviation is defined by the formula:

$$\text{Standard deviation} = \sqrt{\frac{\sum_{i=1}^{n} \left(w_i \left(Y_{cal_i} - Y_{obs_i}\right)\right)^2}{DOF}}$$

where $n$ is the number of points, $w_i$ are the weights applied to each point and DOF is the number of degrees of freedom for the problem. The number of degrees of freedom is equal to the number of data points minus the number of fitted parameters.

## *S6. Fed-batch reactor scheme*

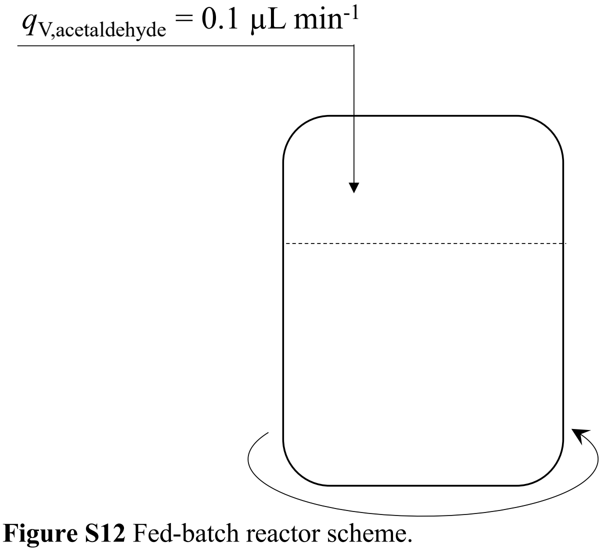


**Figure S12** Fed-batch reactor scheme.

## *S7. Raw material cost calculation*

Annual raw material costs were calculated for the chemical synthesis of the protected alcohol, starting from 3-aminopropanol to obtain *N*-(3-hydroxypropyl)-2-phenylacetamide. Additionally, the annual costs of the nicotinamide coenzyme $NAD^+$ and the enzymes (ADH and NOX) needed for the oxidation of *N*-(3-hydroxypropyl)-2-phenylacetamide **1** to *N*-(3-oxopropyl)-2-phenylacetamide **2** were calculated.

The annual price for 3-aminopropanol, phenylacetyl chloride, $Na_2CO_3$ and $NAD^+$ was calculated according to Eq. 5, for methyltetrahydrofuran and ethyl acetate according to Eq. 6 and for enzyme extract (ADH and NOX) according to Eq. 7. The costs of citric acid, $NaHCO_3$ and NaCl were neglected in this preliminary calculation since the chemicals have low prices. Raw material costs (RMC) per year were calculated as the sum of individual annual chemicals costs.

$$P_{\text{annual chem 1}} = c \cdot M \cdot V_{\text{batch}} \cdot n_{\text{batch}} \cdot P_{\text{chem}} \tag{5}$$

$$P_{\text{annual chem 2}} = \rho \cdot \varphi \cdot V_{\text{batch}} \cdot n_{\text{batch}} \cdot P_{\text{chem}} \tag{6}$$

$$P_{\text{annual enzyme}} = \gamma \cdot V_{\text{batch}} \cdot n_{\text{batch}} \cdot P_{\text{enzyme}} \tag{7}$$

In previous equations, $c$ is the molar concentration of the chemical, $M$ molar mass, $V_{\text{batch}}$ is the volume and $n_{\text{batch}}$ the number of batches per year. $\rho$ is the density of the chemical and $\varphi$ the volume percentage of the chemical in the process. $\gamma$ is the mass concentration of the enzyme. $P_{\text{chem}}$ represents the chemicals retail prices (€ $g^{-1}$) quoted by the suppliers divided by 10 (Tufvesson et al., 2011). $P_{\text{enzyme}}$ represents the price of the enzymes ADH and NOX. Price for ADH was calculated in the same manner as for the chemicals, retail prices (€ $g^{-1}$) were divided

by the factor of 10. The price of NOX was assumed to be 2.5 € $g^{-1}$, according to the literature (Tufvesson et al., 2013; Tufvesson et al., 2011).

### *S8. Synthesis and characterization of N-(2-((2R,4R)-4,6-dihydroxytetrahydro-2H-pyran-2-yl)ethyl)-2-phenylacetamide (5)*

*N*-(2-((2*R*,4*R*)-4,6-dihydroxytetrahydro-2*H*-pyran-2-yl)ethyl)-2-phenylacetamide (**5**) was synthesized starting from *N*-(3-oxopropyl)-2-phenylacetamide (955 mg, 5 mmol, 0.5 M final concentration in the reaction) in 10 mM sodium phosphate buffer pH 7.0. Then, DERA062 (316 mg, 60 U, 6 U $mL^{-1}$ in the reaction) dissolved in 10 mM sodium phosphate buffer pH 7.0 was added. The reaction was initiated by stepwise addition of commercial pure acetaldehyde (**4**) (70.2 μL, 1.25 mmol each 1 h during 8 h, total amounts: 561.2 μL, 10 mmol, 1 M). After 24 h (conversion 90 %), the reaction mixture was diluted with EtOH (100 mL). The mixture was then filtered through Celite®, the pellet washed with EtOH (3 x 50 mL) and the solvent of the filtrate removed under vacuum. The residue was loaded onto activated charcoal (50 mL), packed in a plastic chromatography column (Thomson Instrument Company, 9452090-10, 20x1.9 cm), equilibrated with water and external pressure was applied by compressed air or $N_2$. Impurities were washed with distilled $H_2O$ (3x50 mL). The product was eluted by a stepwise gradient from 5:95 to 100:0 EtOH/$H_2O$ (i.e., twelve steps of 10 mL, increasing by 5 % of EtOH in each step). Fractions containing the product were pooled, concentrated to 20 mL under reduced pressure, frozen at − 80 °C, and lyophilized to yield *N*-(2-((2*R*,4*R*)-4,6-dihydroxytetrahydro-2*H*-pyran-2-yl)ethyl)-2-phenylacetamide (**5**) as a white hygroscopic solid (1.0 g, 71% isolated yield, 5-8% of trimer (**8**) determined by $^1$H NMR). $^1$H NMR (Fig. S16.) (400 MHz, $D_2O$) δ 7.21 (m, 5H), 4.81 (dd, *J* = 10.2, 2.2 Hz, 1H), 4.13 (p, *J* = 3.1x2, 3.0x2 Hz, 1H), 3.71 (m, 1H), 3.43 (s, 2H), 3.20 (dt, *J* = 14.4, 7.3x2 Hz, 1H), 3.10 (m, 1H), 1.74 (m, 1H), 1.52 (m, 2H), 1.36 (m, 3H). $^{13}$C NMR (101 MHz, $D_2O$) δ 174.51, 135.04, 128.90, 127.20, 91.87, 68.26, 64.62, 42.46, 37.98, 36.32, 35.70, 33.79.

## *S9. HPLC chromatograms and NMR spectra*

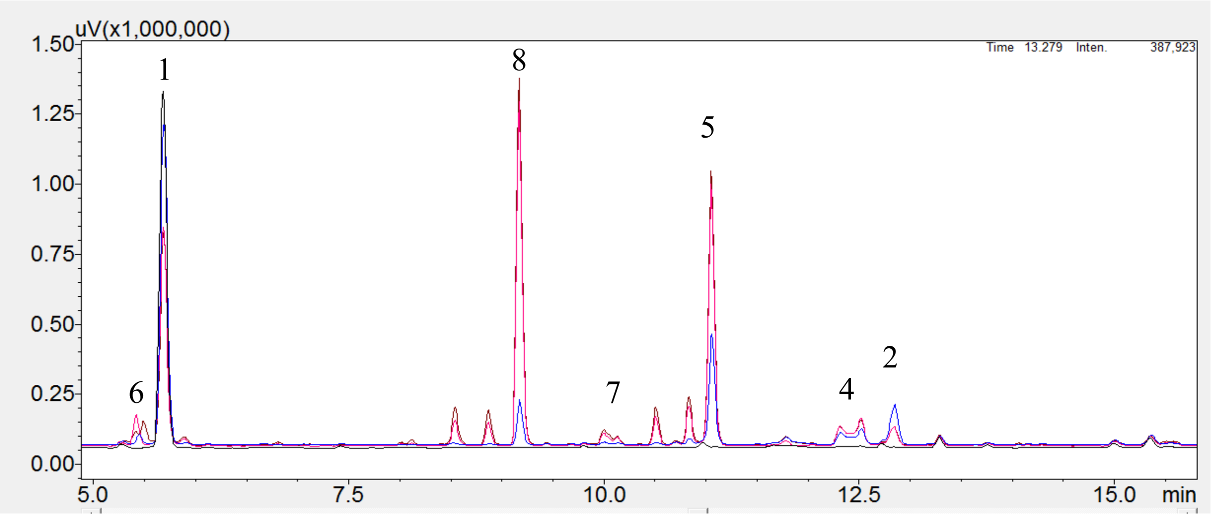


**Figure S13** HPLC chromatograms (acid **6** (RT 5.4 min), alcohol **1** (RT 5.7 min), trimer **8** (RT 9.3 min), dimer **7** (RT 10.1 min), phenylacetamide-lactol **5** (RT 11.2 min), acetaldehyde **4** (RT 12.5 min) and aldehyde **2** (RT 13.0 min)).

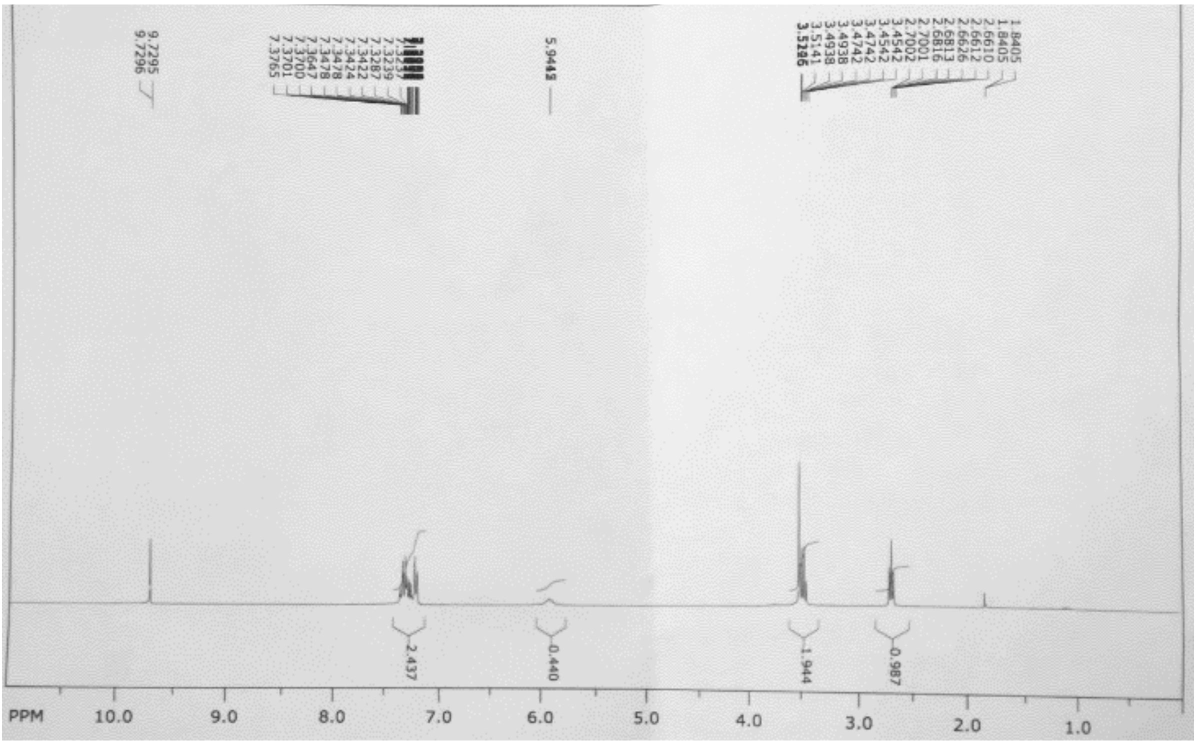


**Figure S14** $^{1}$H NMR of the purified *N*-(3-oxopropyl)-2-phenylacetamide (**2**).

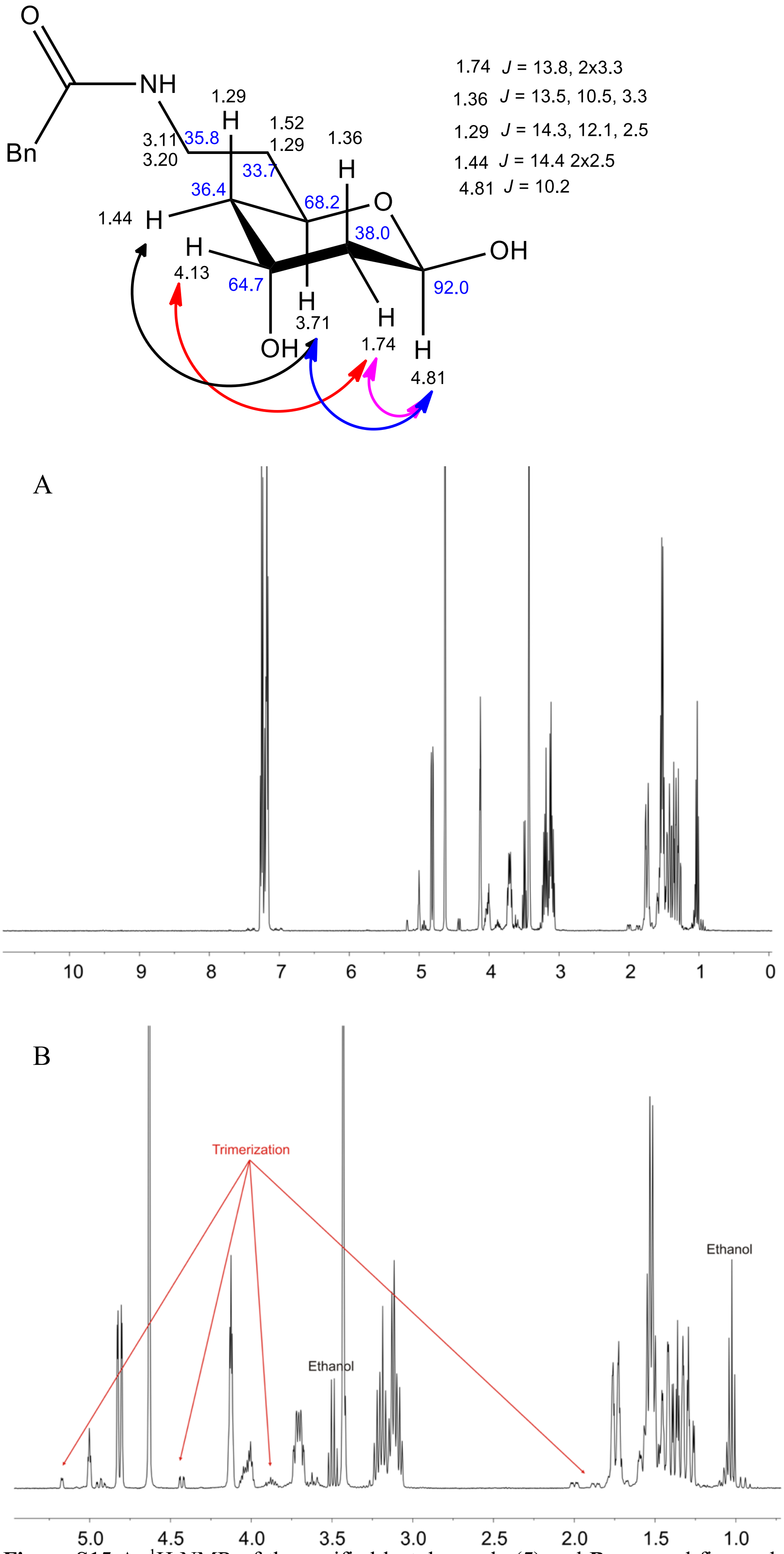


**Figure S15 A**. $^{1}$H NMR of the purified lactol sample (**5**) and **B**. zoomed figure showing traces of side product trimer (**8**) present in the sample.

A

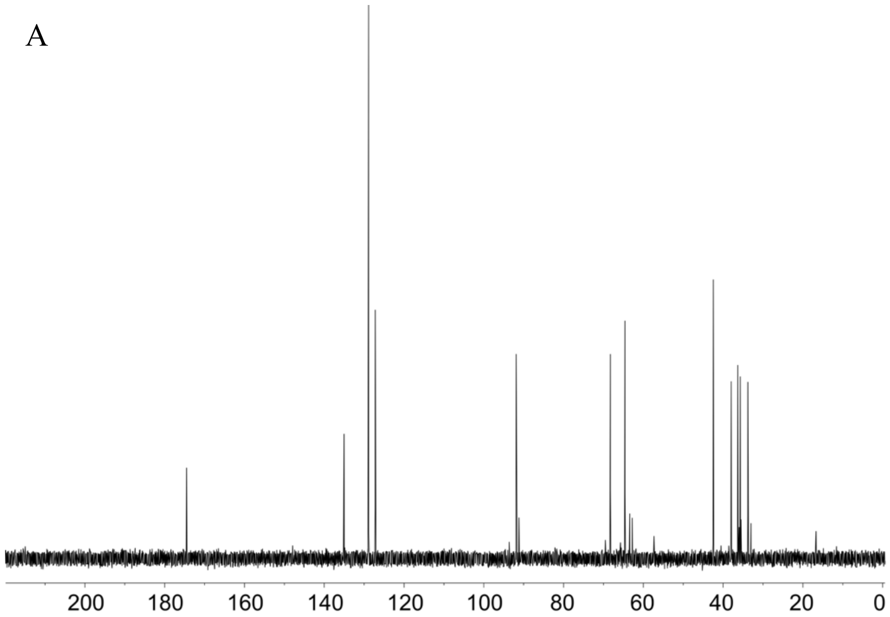


B

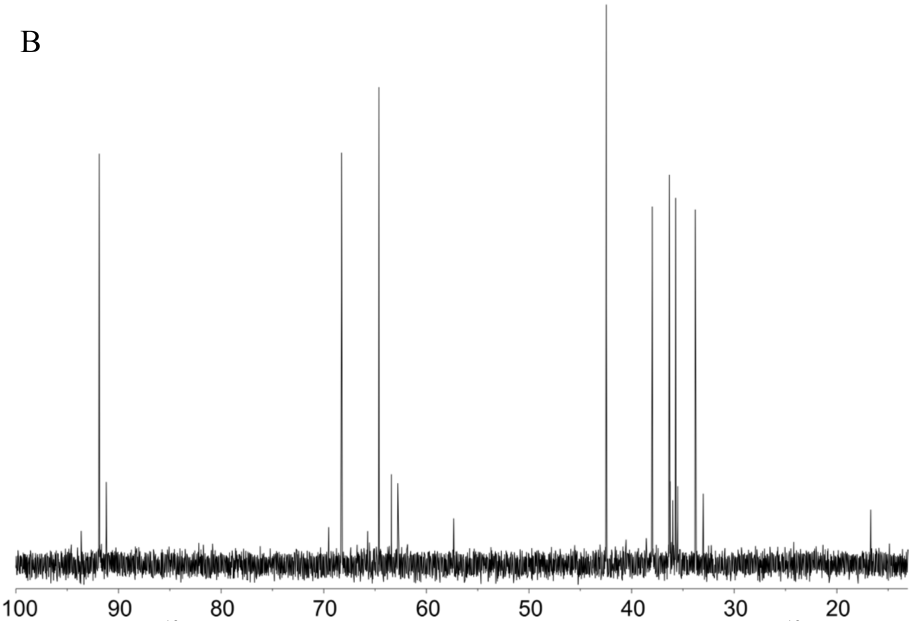


**Figure S16** **A**. $^{13}$C NMR of the purified lactol sample (**5**) and **B**. zoomed $^{13}$C NMR of the purified lactol (**5**).

## References:


Calveras, J; Bujons, J; Parella, T; Crehuet, R; Espelt, L; Joglar, J; Clapés, P; Influence of *N*-amino protecting group on aldolase-catalyzed aldol additions of dihydroxyacetone phosphate to amino aldehydes, Tetrahedron (2006) 62 (11), 2648–2656.

Iley, J; Norberto, F; Sardinha P; The acid-catalysed decomposition of *N*-nitrotolazoline. J Chem Soc, Perkin Trans 2. (1998) (10), 2207–2210.

Tufvesson, P; Lima-Ramos, J; Haque, N A; Gernaey, K V; Woodley, J M; Advances in the process development of biocatalytic processes, Org Process Res Dev (2013) 17 (10), 1233–1238.

Tufvesson, P; Lima-Ramos, J; Nordblad, M; Woodley, J M; Guidelines and Cost Analysis for Catalyst Production in Biocatalytic Processes, Org Process Res Dev (2011) (15), 266–274.
